# Bone-modifying therapy in multiple myeloma: a comprehensive review

**DOI:** 10.3389/fonc.2026.1722230

**Published:** 2026-04-15

**Authors:** Martina Bogeljić Patekar, Vibor Milunović, Inga Mandac Smoljanović, Sandra Bašić Kinda, Slobodanka Ostojić Kolonić, Delfa Radić-Krišto, Slavko Gašparov, Muhamed Baljevic

**Affiliations:** 1 Division of Hematology, Clinical Hospital Merkur, Zagreb, Croatia; 2 School of Medicine in Zagreb, University of Zagreb, Zagreb, Croatia; 3 Division of Hematologic Malignancies, University Hospital Zagreb, Zagreb, Croatia; 4 Clinical Department of Pathology and Cytology, Clinical Hospital Merkur, Zagreb, Croatia; 5 Department of Medicine, Vanderbilt University Medical Center, Nashville, TN, United States

**Keywords:** biosimilars, bisphosphonates, bone pathogenesis, denosumab, multiple myeloma, osteolysis

## Abstract

Multiple myeloma (MM) is a clonal hematologic malignancy characterized by plasma cell proliferation in the bone marrow. One of the most common clinical presentations is skeletal-related events (SREs), characterized by osteolysis of the bone, leading to a significant morbidity and mortality. The main aim of this review article is to provide an overview of the pathogenesis and evidence-based, guideline-recommended treatment of SREs. MM bone homeostasis is altered by numerous pathways and cytokines, leading to the pathogenesis of bone and osteolytic lesions. Historically, bisphosphonates were the mainstay of treatment, but due to toxicities and contraindications in renal impairment, denosumab—a monoclonal antibody targeting the RANKL-RANK axis, given once per month—may have become the treatment of choice, especially with three biosimilars being approved, yet adverse events may be troublesome. Furthermore, it seems that anti-MM-directed therapy has an impact on bone turnover, yet the results are premature. However, despite basic research unraveling novel targets such as micro ribonucleic acids, translational research and randomized clinical trials are lagging behind, making this area an unmet need—despite the fact that precision, risk-adapted, biomarker- and imaging-driven medicine should be a valid clinical goal in this setting.

## Introduction

Multiple myeloma (MM) is a hematological malignancy characterized by abnormal proliferation of plasma cells in the bone marrow and is usually associated with increased bone pain and skeletal-related events (SREs), such as pathological fractures and/or spinal cord compression (1, 2). The incidence rate in the U.S. is 7.2 cases per 100, 000 men and women, with 5-year overall survival (OS) being 61.1% (3). It is important to note that MM is a disease of the elderly. The incidence of MM is rising, especially, in developed countries, with it being the 14^th^ most common malignancy (4). MM presents with bone disease in approximately 80% of cases, anemia in 60%, hypercalcemia in 30%, and renal involvement in approximately 20% of patients (5). Myeloma bone disease results in changes in the bone marrow microenviroment, manifested by increased osteoclastic activity and/or decreased osteoblastic activity, which negatively affects quality of life, causes significant morbidity, and poor outcomes in terms of OS (6). In a study by Terpos et al. on 510 subjects, early SREs were associated with poor OS in multivariate analysis (RR for death = 2.46) (7). SREs, i.e., osteolytic lesions of the skeleton, are a hallmark of MM, with the majority of patients developing SREs (50–60%) during the course of the disease (8). In the IMAJEM study, the authors evaluated magnetic resonance imaging (MRI) and positron-emission tomography-computed tomography (PET-CT) in 134 subjects treated with lenalidomide, bortezomib, dexamethasone (RVd) induction therapy, autologous stem cell transplant (ASCT) if eligible, and lenalidomide maintenance therapy (9). There was no statistical difference between the detection of SREs by imaging modality, while end-of-induction PET-CT negativity was a significant predictor of progression-free survival (PFS) (HR for event = 0.419), unlike MRI, suggesting that PET-CT has prognostic value. However, the International Myeloma Working Group (IMWG) suggests that whole-body low-dose computed tomography is the standard of care (SOC) at MM diagnosis, while other modalities (MRI, PET-CT) can be use in inconclusive cases (10).

As seen above, SRE is a crucial element of MM. The main aim of this review is to describe the pathogenesis and current treatment of SREs in this population, with future perspectives.

### Skeletal homeostasis

Skeletal homeostasis is a dynamic process of interaction between the bone matrix, osteoclasts, osteoblasts, osteocytes, and the metabolic and immune systems, regulating various cellular pathways (11). Osteoblasts arise from bone marrow mesenchymal cells, a process regulated by multiple pathways, and are primarily responsible for bone formation, mainly via secreting various extracellular proteins such as osteopontin and osteocalcin. After bone formation, the majority of these cells undergo apoptosis, differentiate into osteocytes (cells with osteoclastic potential), or become dormant (12–14). The second type of cells, with bone resorption potential, are osteoclasts, which arise from monocyte and macrophage cells (15). Physiological bone remodeling is defined by bone formation and bone resorption, which is a dynamic process in the bone microenvironment, with the dominant pathway involving a member of the receptor activator of nuclear factor kappa-B (RANK) and its ligand (RANKL), initially discovered and reported in lymphocyte interactions (16). RANK/RANKL pathways regulate the differentiation of osteoclasts, as shown by *in vivo* murine models of suffering from osteopetrosis (17, 18). We must note that recent research has defined two more types of cells involved in the skeletal homeostasis. The first type of cell is the bone lining cell, by definition an osteoblast differentiation product covering the surface of the bone (19). These cells are positioned in areas where there is no resorption or bone formation. Yet, they can differentiate into osteoblasts via the parathyroid hormone (PTH) pathway. In murine models, another type of cell involved in bone homeostasis has been identified, i.e., osteomorphs (20). By using single-cell resolution, osteomorphs were identified as a distinct type of osteoclasts. They are formed by the fission of osteoclasts, but also can fuse with these cells. Osteomorphs are under the direct control of RANKL and have a distinct genotype when compared with osteoclasts and their precursors, and have recently been associated with bone homeostasis in humans through genome-wide analysis in the UK Biobank (21). The detailed description of their role is out of scope of this review article, and while these insights have great translational value, our understanding of these processes is in its beginning.

Another important glycoprotein in the homeostasis of the bone is osteoprotegerin (OPG), a member of the tumor necrosis factor (TNF) family, inhibiting the formation of osteoclasts (22). Any imbalance between these processes can lead to impaired bone homeostasis. Under normal circumstances, bone remodeling is dynamically balanced between bone resorption by osteoclasts and new bone formation by osteoblasts. Impairment of this complex system of cellular pathways and the interplay of osteocytes, osteoblasts, and osteoclasts is implicated in multiple diseases such as osteoporosis, rheumatoid arthritis, and solid cancer metastases (23). It is important to note that bone homeostasis is beyond the scope of this review article (for further reading, see Leszczyńska et al., 2025) as we will focus on MM and SREs (24).

### The pathogenesis of multiple myeloma skeletal-related events

The pathogenesis of SREs in MM is a complex interplay of various cells, intracellular pathways, and cytokines (25). Although reduced in the bone of MM patients, osteocytes induce the activity of osteoclasts via the Notch 3 and 4 pathways and receptors, and active RANKL and OPG, leading to bone resorption and loss (26). Furthermore, malignant MM cells interact with the bone marrow environment via CD49d, leading to bone resorption and disease pathogenesis (27). The RANK/RANKL/OPG pathway is activated, leading to high levels of the soluble ligand and low levels of OPG, resulting in the activation of osteoclasts and SREs in MM (28). It is important to note that the RANKL/OPG ratio may be prognostic in terms of OS (29). Various cytokines, such as macrophage inflammatory protein-1α, activin A, interleukin-3, interleukin-6, and tumor necrosis factor α, simulate osteoclasts via various cellular pathways, leading to SREs (30). On the other hand, in MM, osteoblast formation is inhibited by various pathways and paracrine modulation, leading to impaired bone formation (31, 32). The canonical Wnt pathway, responsible for osteoblast creation and survival, is inhibited by various paracrine inhibitors, such as Dickkopf-1, secreted by MM or bone marrow stromal cells (BMSCs) (33, 34). Various cytokines, such as interleukin-3, produced by MM cells, inhibit osteoblast differentiation (35). Bone resorption activates members of the tumor growth factor-β family, which suppress bone formation and are linked with poor outcomes despite lenalidomide-based therapy (36, 37). In conclusion, bone homeostasis is altered in MM, promoting bone resorption and, therefore, SREs.

## Treatment and prevention of multiple myeloma skeletal-related events

Various treatment strategies are available for patients with MM and myeloma bone disease (MBD). Controlling the underlying MM with triplet or quadruplet therapy, ASCT if eligible, and consolidation or maintenance is vital, as MBD will either develop or worsen without proper management of the primary disease (38). Additional options may include radiotherapy for massive SREs or surgical intervention (39, 40). Preventative treatments are essential to slowing the progression of MBD. At present, antiresorptive agents remain the gold standard of preventative therapy. These approaches, often used in combination, aim to address the complex challenges of SREs (41). Concerning these approaches, International Working Group for Multiple Myeloma (IMWG) guidelines recommend in case of severe pain due to spinal cord compression or other neurologic symptoms, often related due to extra nodal disease, alternative approaches, such as radiation or surgical decompression alongside antiMM therapy (42). In case of no improvement, the cemental augmentation or balloon kyphoplasty are treatments of choice to restore spinal stability.

## Bisphosphonates

Bisphosphonates (BP) are pyrophosphate analogs that bind to hydroxyapatite at bone turnover sites. Osteoclasts absorb BPs, inhibiting farnesyl pyrophosphate synthase and reducing osteoclastic activity, thereby preventing bone destruction (43). It is important to note that they are divided into two groups based on their chemical structure, i.e., the presence of nitrogen. The first in the class of BPs were clodronate and etidronate, which lack the nitrogen compound (44). While etidronate use had no effect on SREs in RCTs when compared with placebo, clodronate was tested in the VIth MRC Multiple Myeloma Trial on 536 subjects randomized to the experimental arm (clodronate 1600 mg per os) or placebo (45, 46). The incidence of non-vertebral fractures (p < 0.01) and vertebral fractures (p = 0, 0012) in patients with already documented MBD was significantly lower in the experimental arm when compared to placebo. Subjects without MBD at study entry experienced fewer SREs in the clodronate arm. Another Finnish RCT on 350 subjects showed fewer osteolytic lesions in the clodronate group (12% vs. 24%, p = 0.026) (8, 47). However, the authors did not find a statistically significant difference in vertebral fractures incidence (30% vs. 40%). Despite being potent in MM SREs, the usual use of this compound is limited to the treatment of osteoporosis in postmenopausal women, and its use is limited in MM due to novel agents (48). The nitrogen-containing BP, i.e., pamidronate, was tested on 392 patients, with the control arm being placebo in advanced stage III MM cases (49). Pathological fractures in the experimental group were less common in the pamidronate group compared to placebo (17% vs. 30%, p = 0.006), as well as the need for palliative radiotherapy (14% vs. 22%, p = 0.05). Furthermore, quality of life (QoL) and pain control were preserved in the pamidronate arm. Long-term follow-up was published after 21 cycles of pamidronate with anti-MM therapy (50). The mean incidence of SREs was lower in the pamidronate group compared to placebo (1.3 vs. 2.2, p = 0.008). In a meta-analysis including 11 trials on clodronate and pamidronate (N = 1113), a reduction in vertebral SREs (OR = 0.59, 95% CI 0.45–0.78, p = 0.0001) and improved pain symptoms (OR = 0.59, 95% CI 0.46–0.76, p = 0.00005) was noted, without significant heterogeneity among data (51). In terms of the number of patients needed to treat, 10 patients treated with BPs are needed to prevent one vertebral SRE and 11 to prevent pain symptoms, indicating that at the time of the data cut-off, currently available MM bone-modifying drugs are not satisfactory. Furthermore, no reduction in non-vertebral SRE incidence was seen, representing an unmet need in this group of patients. The most potent BP is zoledronic acid (52). The most important RCT on MM and SREs, the so-called “MRC Myeloma IX,” compared zoledronic acid to clodronate in 1, 960 newly diagnosed MM patients treated with cyclophosphamide, thalidomide, and dexamethasone or cyclophosphamide, vincristine, doxorubicin, and dexamethasone, with the incidence of SREs being a secondary end point (53, 54). The majority of subjects prior to entry into the trial had MBD documented by plain skeleton radiography. Zoledronic acid was shown to be superior to clodronate regardless of chemotherapy arm in terms of new skeletal lesions (27% vs. 35%, HR = 0.74, 95% CI 0.62–0.87, p = 0.0004). The incidence of SRE-related morbidity was lower in the zoledronic group (0.4 vs. 0.8 events per patient per year). However, no difference in radiotherapy or surgery was noted. The main criticism of this trial lies in the fact that plain radiography was used, underscoring the extent of bone disease. Concerning pamidronate, Sanfillipo et al. performed a retrospective study on 1, 018 U.S. veterans, showing the superiority of zoledronic acid with 25% reduction in SREs (HR = 0.75, 95% CI 0.60–0.94) (55). An updated Cochrane meta-analysis on BP use included 20 RCTs (N = 6, 692) on MM patients (56). When compared with placebo, their use prevents vertebral fractures (RR = 0.74), other SREs (RR = 0.80), and results in better pain control (RR = 0.75), supporting their use in everyday practice in the treatment of MM. It is important to note that zoledronic acid was not superior to all other commonly used BPs except etidronate. The true clinical and practical value of zoledronic acid lies in the infusion duration of only 15 minutes, which supports better compliance and persistence compared to oral BPs, as shown in a meta-analysis of 83 studies (HR for treatment drop-out = 0.73) (57). This finding has clinical importance, as patients not taking bisphosphonates as recommended are at increased risk for SREs. The studies described above are summarized in **Table 1**.

**Table 1:** Bisphosphonates in the treatment of multiple myeloma.

Trial	N	Compound	Comparator	SREs	Comments
Belch et al. (45)	173	etidronate	placebo	N.S	MP regimen
McCloskey et al. (46)	536	clodronate	placebo	15% vs 29%^1^ 41% vs 60%^2^	Various anti-MM protocols
Lahtinen et al. (47)	350	clodronate	placebo	24% vs 54%^3^	MP regimen
Berenson et al. (49)	392	pamidronate	placebo	24% vs. 41%^4^	First and second line therapy
Berenson et al. (50)	392	pamidronate^5^	placebo	1.3 vs 2.2 ^6^	First and second line therapy
Terpos et al. (51)	1960	zoledronic acid	Clodronate	27% vs. 35%^7^	Newly diagnosed MM

N, number; SREs, skeletal-related events; MP, melphalan, prednisone; MM, multiple myeloma.

1. non-vertebral fractures.

2. vertebral fractures.

3. progression of lytic lesions, no significant incidence of vertebral fractures.

4. events defined as pathological fracture, irradiation, spinal decompression; improvement in quality of life.

5. 21 cycles of pamidronate.

6. events defined as in first analysis, events per year.

7. primary outcome SRE, secondary outcomes: fewer vertebral and other fractures, novel osteolytic lesions, X-ray used as defining outcomes, first report of study focused on outcomes in terms of survival, questionable benefit of zoledronic acid over pamidronate and clodronate as outlined by Mhaskar et al. (56).

### Can bisphosphonate modify the multiple myeloma disease course?

BPs also exhibit direct anti-myeloma and immunomodulatory effects (25). *In vivo* murine models show osteocyte apoptosis, changing the microenvironment and promoting homing and replication of the MM clone (58). This process is naturally inhibited by BPs (59). MM cells express very late antigen-4, interacting with BMSCs and leading to homing and malignant clone proliferation, which can be inhibited by bone resorptive inhibitors (60). MM cells-microenvironment paracrine interaction can also be inhibited (61). These and other studies serve as a biological basis of bisphosphonates to alter the course of MM when used in combination with anti-cancer MM therapy (62, 63). Concerning clodronate, after a follow-up of 5 years, McCloskey et al. did not show an OS advantage in patients presenting with vertebral fractures at the commencement of the study (p = 0.22), but subjects without preexisting SREs had better outcomes in terms of OS when compared to placebo (HR = 0.62, 95% CI 0.43–0.62) (63). Despite OS not being reported by investigators in the Finnish clodronate study, MM-specific outcomes in terms of progression of SREs—defining relapsed/refractory disease per CRAB criteria—were twice as high in the placebo group as in the clodronate group (24% vs. 12%, p = 0.026) (47). Despite not showing an OS benefit in first-line treatment, pamidronate showed an OS benefit in relapsed/refractory MM patients receiving second-line treatment (21 vs. 41 moths, p = 0.046), although the basis for these findings remains unclear (48). In a U.S. retrospective study on zoledronic acid and pamidronate, better OS was observed in the zoledronic arm, with a 22% reduction in death in multivariate analysis (HR = 0.78, 95% CI 0.67–0.92, p = 0.002) (56). Long-term follow-up of the MRC Myeloma IX RCT, spanning 72 months, showed better PFS (19 vs. 18 months, HR = 0.89, 95% CI 0.80–0.92, p = 0.02) and OS outcomes (52 vs. 46 months, HR = 0.86, 95% CI 0.77–0.97, p = 0.02) in the zoledronic acid group when compared to clodronate, after adjustment for competing factors (54). Yet, we must note that the effect size and clinical significance are questionable due to the high HRs and broad confidence intervals, which cast doubt on the relevance of these findings. A Cochrane analysis on BPs showed marginal and low-evidence for a PFS effect when compared with placebo in 908 patients (HR = 0.75, 95% CI 0.57–1.00, p = 0.05) (64). Concerning PFS, when comparing BPs (N = 3427), no statistically significant beneficial effect of zoledronic acid over clodronate was noted (HR = 1.10, 95% CI 0.46–1.95). Yet, in terms of OS, outcomes were improved when compared to placebo (HR = 0.67, 95% CI 0.46–0.91) and etiodronate (HR = 0.56, 95% CI 0.29–0.87).

### What is the optimal duration and dosage of bisphosphonates in the various stages of multiple myeloma?

This issue is still a matter of debate among experts (65). The Nordic Myeloma Study Group performed an RCT on the dosage of pamidronate (90 mg vs. 30 mg) for three years (66). The primary outcome was physical function at 12 months, assessed by the EORTC QLQ-C30 questionnaire, in 504 MM subjects (67). There was no difference concerning dosage in the primary endpoint (p = 0.52), nor in the full follow-up (p = 0.88). Regarding SREs, there was no difference among the groups (p = 0.32), nor in time to event (p = 0.32). A CALGB non-inferiority phase III RCT tested different zoledronic acid schedules (1 vs. every 3 months) in 1, 822 subjects with metastatic breast or prostate malignancies and MM (N = 278), comparing the incidence of SREs within two years of follow-up (68). In the MM group, the 3-month dosing schedule was not inferior to the 1-month schedule (mean difference = 0.05, p = 0.14). However, the biomarker C-telopeptide was lower in the 1-month treatment group (p = 0.05), indicating biologically better bone homeostasis. No benefit from prolonged monthly zoledronic acid treatment (4 years) was shown in MM patients concerning SREs (N = 93, HR = 1.02, p = 0.9) in an observational study by Van den Wyngaert et al. (69). Yet, despite not showing improved outcomes in terms of OS and PFS, an RCT on the prolonged use of zoledronic acid in MM patients undergoing ASCT (N = 170) showed a lower incidence of SREs in the experimental group (21% vs. 41%, p = 0.01) (70). Similarly, the Nordic Magnolia study confirmed these findings with efficacy and no new safety signal (71). Furthermore, a risk-adapted, biomarker-driven RCT (based on urinary N-telopeptide of type 1 collagen [uNTX]) was performed on 121 patients already treated with BPs to assess the effects of prolonged use of these agents (72). Due to low uNTX levels, the majority of patients (N = 117) began receiving zoledronic acid every 3 months, but crossover to monthly infusion was significant (N = 38), mostly due to progressive disease. Regarding the incidence of SREs, this RCT showed reduced rates in the once-monthly group (p = 0.049). However, 59 patients discontinued the study, mostly due to adverse events (AEs) (N = 6) and withdrawal of consent (N = 52), with a minority of patients completing the full protocol—indicating that this prolonged use is not feasible in everyday life.

We believe that first-line treatment of MM with bisphosphonates should be standard of care until the patient achieves VGPR to primary anti-MM therapy. While, the dosing schedule and duration revolves around 12–24 mo period, though freedom is left for personalized administration plan which is our preferred approach with every patient. Based on patient- and disease-related factors, and tested preferably in academic RCTs. Regarding the relapsed setting, we recommend a similar approach.

## Side effects of bisphosphonate therapy

Concerning BPs, one of the AEs is a spectrum of renal (AEs) (73). Large-scale data from the FEARS U.S. database reported 52, 495 AEs associated with zoledronic acid, of which 17, 936 were in MM patients and linked to renal pathology. For the entire group, the authors recognized 25 distinct renal disorders associated with zoledronic acid. In MM patients, proteinuria was the leading AE (ROR = 6.84), followed by tubulointerstitial nephritis (ROR = 6.59) and renal tubular necrosis (ROR = 5.57). The rate of AEs leading to hospitalization was 19.7%, with a TRM rate of 8.8%. Yet, this MM database contains a certain bias, as MM progression itself presents with various renal pathological states, especially symptomatic or asymptomatic proteinuria or renal insufficiency (74, 75). Furthermore, BPs are contraindicated in patients with a mean glomerular filtration rate (MGFR) less than 30mL/min/1.73 m^2^. One of the most serious AEs of bisphosphonate therapy is medication-related osteonecrosis of the jaw (MRONJ) (76). A single-institution longitudinal cohort study by Oloffson et al. included 144 MM patients and examined oral health status during the disease course. The incidence of ONJ was 2.5 per 100 persons-years, with risk factors in multivariate analysis including missing teeth, ≥3 lines of systemic therapy, periodontitis, and undergoing a dental procedure after BPs were introduced. The most clinically relevant risk factor was dental procedure (RR = 7.75, 95% CI 7.14–8.36), indicating that, prior to introducing BPs in MM care, referral to a stomatologist represents good clinical practice. We must note that steroid use, the mainstay of anti-MM regimens, is an important factor in this setting. Furthermore, a recent systematic analysis examined awareness of MRONJ among 10, 536 dentists and 318 patients (77). Despite being aware of MRONJ as a possible entity, only 9.7% of dentists were aware of risk factors representing a lack of good clinical practice. The results among the patients were more dismal, with only 19.5% being aware of this AE, indicating the lack of properly informing this vulnerable population. It is optimal approach that the managing MM providers (physicians, advanced care providers etc.) should optimally inform and counsel patients on MRONJ, risk factors, and the need for good oral hygiene. Another important AE is atypical femur fracture occurring mostly in subtrochanteric region. Black et al. estimated the incidence in postmenopausal women receiving BP (oral or intravenous) as 1.74 fractures per 10, 000 patient-years, with the greatest risk factor being BP exposure duration per year and the use of steroids (78). Swedish registry data on 787 cancer or osteoporosis patients reported AEs related to bisphosphonate use, including atypical femur facture in 55 cases, commonly associated with prolonged use of these agents (79). These data suggest that “BP therapy” holidays may be of importance in this population, which may translate into MM patients. Yet, a meta-epidemiological study has shown a high risk of bias in reporting MRONJ and atypical femoral fracture, leading to overestimation of these AEs (OR for MRONJ = 1.48, OR for atypical femur fracture = 1.85) (80). A Cochrane meta-analysis by Mhaskar et al. investigated AEs of various BPs (56). Concerning MRONJ, RR was 4.61, though the 95% CI was wide (0.99–21.35), and the quality of evidence was low. In further analysis of RCTs and observational studies, the main conclusion was that 1, 000 patients would need to be treated with BPs for one occurrence of MRONJ, which is in line with previous reported data by Guegeon et al. (79). Updated Cochrane analysis by Mhaskar et al. showed similar risk for MRONJ, although 8 RCTs reported this AE, while observational studies differed in reporting MRONJ (0%–51%), indicating inconsistent reporting (64). BPs may cause electrolyte disturbances, primarily hypocalcemia and hypomagnesemia (81). Calcium and vitamin D supplementation is recommended, but in a retrospective study of 120 patients treated with zoledronic acid—mainly suffering from MM—hypocalcemia requiring intravenous correction occurred in 8% of subjects, leading to a decrease of renal function. It is important to note that this AE can result in lethal outcomes as described by Duval et al. in a single-center study (82). Another AE of interest is secondary hyperparathyroidism (83). Elevated parathyroid hormone (PTH) promotes osteoclast activity, thereby increasing the risk of SREs. Moreover, PTH may be implicated in tumor progression due to its effects on cytokines, the bone marrow environment, and malignant clone behavior. We must note that this AE is often overlooked, and PTH levels should be monitored during BP treatment and managed appropriately. Other AEs are less clinically significant, such as self-limited acute phase responses in patients receiving intravenous BPs, or gastrointestinal discomfort in those taking BPs orally (84). Contrary to other data, Mhaskar et al. did not find a difference in the incidence of hypocalcemia between BPs and placebo, which may be attributed to biases in its monitoring in RCTs (56). There was also no difference in renal insufficiency between intravenous BPs and placebo, yet based on earlier data, pamidronate and zoledronic acid carry a Food and Drug Administration (FDA) black box warning. We must interpret all these findings with caution, given that RCTs and observational studies in this area have multiple biases and a low level of evidence. It seems there is a need, due to conflicting studies, for a large multicenter, prospective academic observational study to capture true AEs of BPs treatment in MM patients. Until then, an evidence-based approach towards these patients will not be possible, and the managing providers still needs to treat AEs of BPs on an individual patient basis.

## Denosumab

Denosumab is a fully human monoclonal IgG2 antibody that inhibits RANKL and competes with its interaction with RANK (84). It inhibits the formation and proliferation of osteoclasts, as well as their function and survival. Unlike BPs, it has a rapid onset of action. It restores bone homeostasis in MM *in vitro* models, and it has been shown that serum N-telopeptide levels—a biomarker of osteoclasts—are reduced in MM patients receiving denosumab (85). It is important to distinguish between the two formulations and different indications of denosumab. The first one (trademark name of the original product “Prolia”) is given every 6 months subcutaneously at a dose of 60 mg for osteoporosis (86). Because a significant proportion of MM patients present with renal insufficiency, and BPs are metabolized through the kidneys and can cause nephrotoxicity (which limits their use), the distinct pharmacokinetics of denosumab have made its use in MM of increasing interest (86). The first non-inferiority RCT examined denosumab treatment on 1, 776 patients mostly suffering from non-small cell lung cancer carcinoma and MM compared to zoledronic acid with the exclusion of breast and prostate advanced neoplasms (87). It is important to note that MM represented a minority of the study population (N = 180). The primary outcome was the first on-study SRE (20.6 months for denosumab, 16.3 months for zoledronic acid, p = 0.06). In the MM population, despite greater suppression of bone turnover markers, there was no difference in the primary outcome (HR = 1.03, p = 0.89). Of AEs of importance was worsening of renal function in zoledronic arm, while expected hypocalcemia was predominant in the denosumab arm. There was no difference in MRONJ events. However, in further analysis, denosumab appeared superior in terms of pain prevention, opioid usage, quality of life, and palliative radiotherapy (88). Concerning long-term outcomes in terms of survival, there was a signal that MM patients receiving zoledronic acid had superior outcomes, but Raje et al., in a *post-hoc* analysis, found multiple confounding factors resulting in similar events, i.e., death, withdrawal of consent, and lost to follow-up (HR = 1.31, p = 0.278) (89). The first large phase III placebo-controlled RCT of denosumab (dosage 120 mg subcutaneously every 28 days) in MM was a non-inferiority trial comparing it to zoledronic acid in 1, 718 newly diagnosed MM patients with MGFR ≥ 30 mL/min/1.73 m^2^, all of whom were treated with bortezomib- and lenalidomide-based therapy (90). The primary endpoint was time to first SRE while on study drugs, documented by skeletal radiography and blind radiologist review. It is important to note that the majority of the population entered the study with MBD. After a median follow-up of 17.3 months, the primary endpoint was met, showing non-inferiority of denosumab (HR = 0.98, 95% CI 0.85–1.14, p = 0.01), with similar rates of SREs (44% vs. 45%) occurring during the first 6 months. Despite being a non-inferiority RCT, *post hoc* analysis showed the superiority of denosumab over zoledronic acid in the occurrence of SREs during the study (HR = 0.66, 95% CI 0.44–0.98, p = 0.039). Concerning long-term outcomes, there was no difference in OS, while the denosumab arm showed improvement in median PFS (46.1 vs. 35.4 months, HR = 0.82, 95% CI 0.68–0.99, p = 0.036). It is important to note that denosumab had a better safety profile in terms of renal AEs. A rise in creatinine from baseline (2 mg/L) was noted in 9% of subjects in the experimental group compared to 16% in the standard group, as well as creatinine doubling (3% vs. 7%). There was no difference in ONJ between groups, yet hypocalcemia was more profound in experimental arm. However, one AE must be noted: the so-called “denosumab rebound effect”—i.e., the worsening of BMD after the discontinuation of denosumab, which may be countered by the use of BPs, especially zoledronic acid. In conclusion, this trial positioned denosumab as an alternative to BPs. In an exploratory analysis of the pivotal denosumab trial, patients proceeding to ASCT, those with normal creatinine clearance, and those younger than 70 years benefited in terms of PFS (91). Otherwise, no benefit was observed in patients with impaired creatinine clearance (≤60 mL/min/1.73 m^2^), those older than 70, and ASCT-ineligible subjects. Regarding induction therapy, denosumab demonstrated better PFS in patients receiving triplet therapy (p = 0.009) and in those receiving bortezomib-based therapy (p = 0.029). In conclusion, these results suggest that denosumab may represent a standard of care in younger patients who are candidates for ASCT and are treated with triplet therapy, such as RVd. Unfortunately, despite denosumab’s non-renal clearance as a monoclonal antibody, there are no formal data in this setting, despite MM often presenting with renal disease. The available data come from cancer and osteoporosis studies, and we may assume that this agent has a better safety profile in the population presenting with bone disease and MGFR less than 30mL/min/1.73 m^2^ (89). In conclusion, we are of the opinion that denosumab will, in the near future, become the standard of care.

The mentioned studies above are shown in **Table 2**. The drugs currently approved by the FDA and EMA for the treatment of SREs in MM in are shown in **Table 3**. As evident, there exist huge disparities among particular healthcare systems, due to reimbursement, out-of pocket costs, and other financial factors, which make MM patients vulnerable with disparate access to denosumab and guideline-recommended management, highlighting this aspect of supportive care as a possible unmet need.

**Table 2:** Denosumab in the treatment of multiple myeloma.

Study	N	Experimental arm	Comparator	SREs	AEs	MM treatment
Henry et al. (87)	1776^1^	Denosumab^2^	Zoledronic acid + placebo	HR = 0.84 p = 0.6^2^	Infections^3^ Rebound effect^4^ Hypocalcemia	various
Raje et al. (90)	1718^5^	Denosumab	Zoledronic acid + placebo	HR = 0.98, p = 0.01^6^	Infections^7^ Rebound effect^4^ Hypocalcemia Safer renal profile in experimental group	Bortezomib or IMID based triplets

N, number; SREs, skeletal-related events; AEs, adverse events; MM, multiple myeloma; HR, hazard ratio; IMiD, immunomodulatory drug.

1. N of MM = 190, most common population non-small cell lung cancer, breast and prostate cancers patients excluded.

2. non-inferiority study, primary endpoint time to SRE, *post-hoc* analysis showed the number of patients to prevent one SRE (3 vs.10 per year), confounding population, better clinically relevant outcomes (87, 88).

3. active treatment, infections, MRONJ, ineligible, hypocalcemia.

4. longer follow-up rebound effect upon cessation of denosumab treatment.

5. first line setting.

6. non-inferiority study, primary outcome first on-study SRE, *post-hoc* analysis showed better outcomes in terms of SREs, better long-term outcomes in transplant-eligible patients (91).

7. infections due to first-line treatment.

**Table 3:** Approved bone‐modifying agents in myeloma bone disease by the FDA and EMA.

Drug		Zoledronic acid	Pamidronate disodium	Denosumab^1^
Drug type		Bisphosphonate	Bisphosphonate	Human IgG2 mAb targeting RANKL
FDA/EMA approved		Yes/Yes	Yes/Yes	Yes/Yes
Indications and usage		Hypercalcemia of malignancy; Multiple myeloma and solid tumor bone metastases	Hypercalcemia of malignancy; Osteolytic bone metastases	Postmenopausal osteoporosis; Glucocorticoid-induced osteoporosis; High fracture risk in nonmetastatic cancers
Dosage and administration		4 mg IV over ≥15 minutes every 3–4 weeks	90 mg IV over ≥2 hours every 3–4 weeks	120 mg SC every 4 weeks
Normal Renal Function		4 mg IV every 3–4 weeks	90 mg IV every 3–4 weeks	120 mg SC every 4 weeks
Mild/moderate reduction in renal function		Reduce dose	Use standard dose	120 mg SC every 4 weeks
Severe reduction in renal function		Not recommended	60–90 mg	120 mg SC every 4 weeks
Warnings and side effects		Renal toxicity; Osteonecrosis of the jaw; Electrolyte disorders - hypocalcemia; Atypical femoral fractures	Renal toxicity; Osteonecrosis of the jaw; Electrolyte disorders - hypocalcemia; Atypical femoral fractures	Osteonecrosis of the jaw; Electrolyte disorders - hypocalcemia; rebound SRE if disrupted
Additional remarks		Dental examination recommended prior to treatment^2^; Supplement with calcium and vitamin D	Dental examination recommended prior to treatment^2^; Supplement with calcium and vitamin D	Renal impairment: no dose adjustment is necessary; Dental examination recommended prior to treatment^2^; Supplement with calcium and vitamin D, application of zoledronic acid in order to prevent rebound effect
Free Market Cost	USA	Zoledronic acid 4 mg/5 ml: 3.60 - 167.66 USD	Pamidronate 60 mg: 5.45 USD	Denosumab (Xgeva) 120 mg/1,7 ml: 2,435.11 USD Denosumab (Wyost) 120 mg/1.7 ml^3^: 2,264.65 USD
	EU representative nation (Croatia)	Zoledronic acid 4 mg/5 ml: 86 €	Pamidronate 60 mg: 73,43 €	Denosumab (Xgeva) 120mg/1.7ml: 271,11 € Denosumab (Wyost) 120mg/1.7ml: 215,66 €

IgG2, immunoglobulin G2; mAb, monoclonal antibody; RANKL, receptor activator of nuclear factor kappa-B ligand; IV, intravenous; SC, subcutaneous; SRE, skeletal-related event; IU, international unit; FDA, U.S. Food and Drug Administration; EMA, European Medicines Agency; €- euro; USA, United States of America; EU, European Union; mg, milligrams; ml, milliliter; USD, United States Dollar.

1. Biosimilars of denosumab have recently been introduced.

2. Clinical data suggests that the initiation of bone-modifying therapy may be safely delayed due to dental work-up.

3. The cost varies based on the specifics of the U.S. healthcare system, and the introduction of biosimilars has not taken effect on the original denosumab price.

## The pitfalls of denosumab treatment—a lesson hardly learned

One of major AEs in the treatment with denosumab is its cessation leading to a rebound effect, i.e., multiple vertebral SREs (92). Upon cessation of treatment, c-terminal telopeptide of type 1 collagen (CTx), a regulator of bone resorption and a biomarker in various bone-related diseases, rises sharply, while bone mineral density declines rapidly, leading to the risk of multiple fractures. One of the pivotal trials describing this AE is the FREEDOM trial and its extension on long-term use of denosumab in osteoporotic women (93). After cessation of denosumab, the risk for vertebral SREs rose to 7.1 patients per year (94). More importantly, in subjects entering the trial with ≥1 vertebral fracture and being treated with denosumab, the prevalence of multiple fractures was greater than in the placebo group (60.7% vs. 38.7%). The odds of these AEs were 3.9 in subjects entering the trial with vertebral fracture, greater than subjects without this event, with a rise of incidence during follow-up. The question posed is how cessation of denosumab affects MM patients, as this is one of the most common features of the disease. Unfortunately, there is a paucity of data in MM, and this phenomenon, despite occurring in everyday clinical practice, must be extrapolated from postmenopausal osteoporosis (43, 95). The European Calcified Tissue Society, in their statement, have summarized possible interventions based on multiple studies in the population of women suffering from osteoporosis, with multiple scenarios based on small clinical trials, observational studies, or case series. In younger patients, denosumab treatment is not routinely recommended, while in short-term denosumab treatment with a low risk of fractures, oral BPs or zoledronic acid are used for a limited time based on bone mineral density or bone turnover markers. However, these scenarios cannot be applied in MM due to the nature of the disease. A realistic scenario is continuous use of denosumab or switching to zoledronic acid 6 months upon denosumab cessation, with bone turnover marker measurement, with zoledronic acid retreatment. Yet, in many countries, bone turnover marker measurement is not routinely available, and an additional dose of zoledronic acid may suffice. In case of intolerance, continuous oral BPs are an option. Yet, the true answer lies in designing an RCT in MM patients to provide a definitive answer to this unmet need, due to the fact that extrapolation is not evidence-based.

Due to the fact that MM causes end-organ damage to the kidneys by various pathological mechanisms, up to 40% of patients are affected, and 10% require dialysis (96). Yet, in the pivotal denosumab trial, subjects with creatinine clearance ˂ 30 ml/min were not included, and the data must be extrapolated from osteoporotic studies (90). Lee et al. have shown in 1, 517 osteoporotic patients with chronic kidney disease transient, but more severe hypocalcemia, especially during the first three months of treatment, hypophosphatemia, and elevation of PTH in their observational study, yet returning to baseline levels at 6 months (97). In a study on female patients with end-stage renal disease, the incidence of very severe hypocalcemia upon denosumab administration, corrected for albumin levels was 10.9% when compared to oral BPs (0.4%), urging aggressive salvage treatment (98). Bird et al. in a large cohort of Medicare-insured female osteoporotic patients treated with denosumab (N = 361, 453), using a target trial emulation method, have shown higher emergency department visits, hospitalizations, and worsening of chronic kidney disease when compared to i.v. or oral BPs (99). Unfortunately, grade V events were recorded, but due to the limitations of the study, the exact incidence of this outcome is unknown. In conclusion, this AE limits the beneficial effect of denosumab and should be a warning for the practicing provider, and in MM patients should be guided by an experienced provider, preferably in consultation with a hematologists. Concerning MRONJ, the FDA data on osteoporotic and cancer patients reported 7, 689 cases of this AE, with the common dose being 120 mg subcutaneously every 4 weeks unlike the osteoporotic dosage (100). Some sequential or concomitant antiresorptive drugs or cancer drugs were associated with a higher incidence of MRONJ. We must note that the greatest effect was seen with the use of steroids, as stated above, which are common drugs in anti-MM regimens.

We must note that atypical femoral fracture is of lesser concern (101). By synthesis of the literature, 31 cases were reported related to denusumab, associated with prior BP treatment, but also with the dosage and frequency, indicating that MM patients are at greater risk.

In conclusion, despite the favorable safety profile in the pivotal trial, real-world evidence shows that hematologists should weigh the pros and cons prior to prescribing denosumab in the prevention of SREs in MM patients (90).

## Imaging of myeloma bone disease

Historically, skeletal X-ray surveys were used in the every-day workup of MM and MBD. Furthermore, the majority of RCTs used this method in their design and were later criticized as a major source of bias in reporting results on SREs due to its low detection rate and the deferral of symptomatic MM. This method is unreliable, reader-dependent, and has low sensitivity and specificity, potentially affecting both diagnosis and treatment (102). Revised IMWG criteria recommend the use of computed tomography (CT)—often low-dose whole-body CT (LDWBCT)—PET-CT, or whole-body MRI (WBMRI) for evaluating patients with suspected MM to detect MBD fulfilling the CRAB criteria of MM (10, 103). Furthermore, more than one focal lesion on whole-body MRI has become an additional diagnostic criterion for MM, based on a study by Hillengas et al. in smoldering MM (sMM), an entity which does not require therapy in everyday practice (104). A total of 149 subjects were included, all undergoing WBMRI. Patients with more than one focal lesion had faster progression to symptomatic MM, with median PFS being only 13 months (HR for progression = 3, 01, p = 0.002). Yet, despite becoming a diagnostic criterion for MM, this imaging modality has its limitations due to MRI availability, procedural complexity, patient discomfort, and other imaging. Recently, new IMWG criteria for MM imaging have recommended LDWBCT (level III recommendation) as the initial step in diagnostic imaging of plasma cell dyscrasia, followed by WBMRI in cases of inconclusive results (10). Concerning PET-CT, it may be an alternative option in symptomatic MM due to its prognostic significance. Zamagni et al. prospectively demonstrated its prognostic value in 192 newly diagnosed patients treated with tandem ASCT after thalidomide-dexamethasone induction (105). In multivariate analysis, extramedullary disease (HR = 5.28) and a standardized uptake value (SUV) greater than 4.2 (HR = 2.13) were adverse factors for inferior PFS. These were the only two parameters associated with inferior OS. Yet, one criticism is that the group received only doublet induction therapy. In the modern era, it has been shown that ASCT may overcome some of the adverse PET-CT factors, as demonstrated in a cohort divided by ASCT eligibility after triplet induction (106). Concerning PET-CT characteristics, only the number of focal lesions greater than three was prognostic of poorer PFS (HR = 2.82) and OS (HR = 2.54), while ASCT was protective in terms of longer PFS (HR = 0.22) and OS (HR = 0.53). Lee et al. proposed a novel prognostic index for ASCT-ineligible patients, combining SUV_max_ ˃ 2.5 and R-ISS, due to the limited ability of R-ISS alone to predict outcomes (107, 108). This index stratified patients into three groups according to OS, yet this new staging system still needs to be validated (109, 110). It is important to note that all other PET-CT parameters, such as the number of focal skeletal lesions, were also prognostic. Concerning WBMRI, it is used to assess different patterns of bone marrow involvement, MM burden, prognosis, and treatment response to bortezomib-based triplet regimens (111). In a large retrospective study by Sun et al. on 180 MM patients divided into high- and normal-risk subgroups, the degree of diffuse marrow infiltration differentiated the groups, while fat fraction was a protective risk factor. The combination of these two parameters distinguished the groups, with the area under the curve (AUC) being 0.732, showing appropriate sensitivity and specificity (112). Regarding SREs, WBMRI has a role in detecting high-risk MM patients, as shown in the Total Therapy trials (113). The presence of three or more focal lesions (defined as the product of perpendicular diameters ≥ 5 cm^2^) was an independent predictor of inferior PFS (HR = 2.7, p ˂ 0.001) and OS (HR = 2.2, p = 0.001). It is important to note that this prognostic factor was independent of R-ISS, gene expression profiling (GEP), and extramedullary disease. In conclusion, imaging in MM has evolved rapidly across all three modalities. Its use is increasingly based on physician and patient preference, the availability of specific techniques, and should be further explored in future trials using risk-adapted therapeutic strategies.

Yet, the question arises about the dose of radiation of LDWBCT and PET-CT. For example, Lee et al. have shown a radiation dose of 2.1 millisieverts (mSv) of the lumbar vertrebra compared to 4.9 mSv on standard CT in trauma patients (114). PET in combination with diagnostic CT has shown a radiation of 24.4 mSv, while PET with low-dose MSCT has a lesser radiation dose, being 14.4 mSv (115). Some may express this imaging carries the risk of secondary tumors, but the median age of MM diagnosis is 69 years according to the American Cancer Society’s Cancer Statistics Center (116). However, the concern of secondary tumors due to radiation exposure is unfounded due to the low incidence, and its pathogenesis is multifactorial, involving MM intrinsic properties and some anti-MM treatment drugs (117).

### Can multiple myeloma treatment have an effect on bone disease?

*In vitro* studies on human MM cell lines have shown that bortezomib has a negative effect on osteoclasts by blocking their maturation through inhibition of the TRAF6 pathway, which is responsible for osteoclast proliferation (118). Furthermore, it stimulates osteoblast proliferation and differentiation, and these data indicate that bortezomib may modulate bone homeostasis (119). Despite several trials demonstrating this effect, they are limited by small sample sizes. The largest experience comes from the pivotal APEX RCT, which showed an increase in alkaline phosphatase (ALP)—a simple biomarker of bone remodeling—in responding patients (120, 121). Unfortunately, the incidence of SREs was not a secondary outcome of the APEX RCT, and the true clinical relevance of bortezomib on MBD remains unknown (120). A *post hoc* analysis of the VISTA study showed less progression to BMD in the VMP arm compared to the MP arm (3% vs. 11%), as well as an increase in ALP (122). Furthermore, ALP levels were associated with ORR to VMP (HR = 3.096, p = 0.001). The levels of Dickkopf-related protein 1 (DKK-1), an inhibitor of the WNT pathway and osteoblast proliferation, were also lower in the experimental group (123). Terpos et al. prospectively explored the effect of VTD consolidation after ASCT on SREs in a group of 42 patients (124). It is important to note that the use of BPs was not allowed per protocol. The pre-transplant rate of SREs was 28.5%, while only one SRE occurred during VTD consolidation, along with alterations in various biomarkers measuring osteoblast activity. These studies may serve as a “proof of concept” that the use of bortezomib—a current standard of care in triplet or quadruplet regimens—can modify MBD. The second-generation proteasome inhibitor carfilzomib (K) targets multiple pathways, leading to the restoration of normal bone homeostasis, based on *in vitro* or *in vivo* studies, such as stimulation of osteogenesis through osteoblast activation via the Wnt pathway (125). In the relatively small, non-interventional CarMMa study (N = 25), which explored Kd doublet therapy without the use of BPs in the relapsed-refractory setting (126), LDWBCT, PET-CT, or MRI was performed prior to Kd treatment, and the majority of subjects (84%) had a new SRE at entry. Kd doublet therapy appeared to reduce these events, with incidence dropping to 28% after a median number of four cycles. This study also explored bone biomarkers, showing increased levels of markers associated with osteogenesis. Yet, the CarMMa study was not powered sufficiently to draw any firm conclusions about carfilzomib’s efficacy in modifying MBD. The third-generation proteasome inhibitor ixazomib (I) promotes bone remodeling through the Sonic Hedgehog pathway (127). In a small investigator-initiated phase II trial (N = 30) using Sodium-18-Fluoride PET-CT and BM biopsy, three cycles of Id were given to r-r MM subjects (128). Concerning the impact of Id treatment on PET, SUV_max_ for particular osteolytic lesions remained unchanged, but the total SUV_max_ of the skeleton was reduced. Yet, despite PET being inclusive in terms of Id treatment, the changes in BM biopsy supported this treatment. Bone structural units after Id treatment were enlarged compared to baseline, with increased trabecular bone volume and other indicators of healthy bone homeostasis. In multivariate analysis, ixazomib was the only predictive factor associated with an anabolic effect on bone formation. This may serve as a “proof of concept” study suggesting that Id treatment affects bone remodeling in MM, though larger future studies are needed to verify these findings. Concerning immunomodulatory agents (IMIDs), lenalidomide (L) and pomalidomide have been shown to be active in restoring bone homeostasis *in vitro* (129). First, it must be noted that their effects are more complex than expected. Firstly, their mechanisms of action are not cytokine-dependent unless given at the highest doses. Furthermore, these compounds do not affect proteins coded by the RANKL-OPG axis, yet they do alter the MM microenvironment. Despite these phenomena, they inhibit osteoclastogenic activity by reducing CD49d and fibronectin interactions with malignant cells. Using GEP, it appears that these compounds act at the DNA level, altering the expression of various genes and their products. To elucidate the mechanism of lenalidomide in bone restoration, Guo et al. identified a possible pathway inhibited in MM pathogenesis—Notch—showing higher expression of related molecules that inhibit osteoclasts (130). Regarding clinical data on the Ld doublet, Terpos et al. retrospectively analyzed 106 patients, showing a reduction in bone resorption in the r-r setting—but not bone formation—only in responders to therapy, as evidenced by reductions in bone resorptive biomarkers (131). When compared to the RVd regimen, it seems that this triplet is more potent, resulting in bone formation without SREs occurring in this subgroup of patients. Yet, the largest trial on RVd was the IFM/DFCI 2009 trial, randomizing 700 subjects to receive either RVd alone or RVd followed by ASCT (132). In *post-hoc* analysis, regardless of imaging modality, the majority of patients had focal lesions upon entry into the study. The strongest predictor of inferior outcome was extramedullary MD (HR for death = 3.894, p = 0.004), while metabolically negative focal lesions before lenalidomide maintenance, assessed by PET, were a favorable risk factor (HR = 0.419, p < 0.01). The anti-CD38 monoclonal antibody daratumumab has shown activity in MBD in a phase II open-label trial involving 57 subjects (133). The main endpoint of this trial was biomarkers of osteoclastogenesis—i.e., C-telopeptide of collagen type 1 (CTX) and tartrate-resistant acid phosphatase 5b (TRACP-5b)—after four months of daratumumab treatment. It is important to note that BP use was permitted per protocol, and the majority of patients had BMD at study entry. In the ITT group (N = 37), the study did not meet its primary endpoint due to the statistical insignificance of CTX and TRACP-5b reduction, likely due to patient withdrawals from progressive disease. Yet, other biomarkers (osteoblast inhibitors), such as DKK-1 and sclerostin, showed significant reductions, suggesting a potential role of daratumumab in bone metabolism. Clinically, the most important endpoint was the incidence of SREs, which did not occur during the treatment period. However, this study has serious limitations, as the majority of patients withdrew from the protocol, and the true value of daratumumab in MBD must be explored more thoroughly.

In conclusion, DRVd, as a standard of care in the majority of countries, might overcome some aspects of MBD, while extramedullary disease remains an unmet need in the treatment of MM. Regarding other agents, there is preliminary evidence of their ability to modify MBD, but these studies are rather “proof of concept” trials and not mature enough to support their use in this indication until RCTs demonstrate their true benefit. We must note these data cumulatively are preliminary with low level of evidence and uncertain clinical potential.

## Guidelines for the treatment of myeloma bone disease

It is important to note that zoledronic acid, pamidronate, and denosumab are FDA-approved agents for the treatment of MBD (134). There are several guidelines on how to use BPs and denosumab in this setting, as shown in **Table 4**, from various recommending bodies: the European Hematology Association–European Society for Medical Oncology (EHA–ESMO), American Society of Clinical Oncology (ASCO), International Myeloma Working Group (IMWG), National Comprehensive Cancer Network (NCCN), and the European Myeloma Network (EMN) (42, 135–138). NCCN guidelines recommend baseline dental examination, assessment of vitamin D status, regular monitoring of renal function (especially with bisphosphonates), and vigilance for osteonecrosis of the jaw. Upon discontinuation of denosumab, maintenance doses of denosumab or a single bisphosphonate infusion are advised to prevent rebound osteoporosis. Denosumab is preferred in patients with renal insufficiency. Patients with creatinine clearance <30 cc/min are at increased risk for severe hypocalcemia, especially with denosumab, and should have calcium levels closely monitored and supplemented appropriately (137). All guidelines recommend a dental work-up prior to starting bone-modifying agents. Invasive dental procedures should be avoided; however, when clinically necessary, BPs or other bone modifying agents should be discontinued for 8–12 weeks before the procedure, and be held for the same period before the next dose after the dental work is completed, with adequate periprocedural antibiotic prophylaxis. Minor dental procedures (e.g. such a teeth cleaning) are not a contraindication for continuing BPs. Concerning the choice of BPs, they are similar, with the exception of EHA–ESMO guidelines, which do not recommend pamidronate due to the fact that zoledronic acid is the most potent BP (level of evidence I, B) (136). Among the guidelines, indications are similar—being newly diagnosed symptomatic or r/r MM regardless of skeletal status. Regarding the duration of BP therapy, a two-year time frame is supported by the majority of guidelines, except the NCCN, which recommends a risk-adapted approach (137). All guidelines recommend regular monitoring of calcium, vitamin D, creatinine, and possible ONJ, while ASCO also includes albuminuria (135). However, it is important to note that these guidelines are outdated. The frequency of BP administration depends on patient response and physician discretion). Supplementation with calcium and vitamin D is required. Regarding the response to anti-MM therapy, in cases of very good partial response or better, BPs may even be omitted. In cases of renal insufficiency, denosumab is the treatment of choice—despite not being formally tested in MM with severe renal impairment (64). It should be given continuously, but if interrupted, due to the rebound SRE SAE, BPs should be initiated. The most comprehensive guidelines come from IMWG (42). The initiation of treatment with BPs (zoledronic acid, pamidronate) is indicated in active MM regardless of the presence of SREs (grade A) with zoledronic acid being superior for malignant hypercalcemia (grade B). All other entities, such as monoclonal gammopathy of undetermined significance, should be managed per local osteoporosis guidelines (grade C). The notable exceptions are high risk smoldering multiple myeloma (SMM) or SMM presenting with focal lesions without the evidence of osteolysis (grade D). Due to compelling evidence that zoledronic acid greatly reduces mortality, it is preferred over pamidronate (grade B). The duration of zoledronic acid is recommended to be 12 months (grade B) or longer, if a very good partial response (VGPR) is not achieved (grade D). In case of VGPR, the reduced dosing frequency or discontinuation of zoledronic acid is based on various factors (patients’ individual risk for fracture, steroid therapy) (grade D). Concerning denosumab, it is indicated in the first line treatment of MM (grade A) or r-r MM with extensive bone disease (grade B). This compound is equivalent to zoledronic acid (grade A), but is preferred in ASCT candidates or patients with renal dysfunction (grade B). Denosumab should be given until unacceptable toxicity (grade A). Discontinuation should be taken into account after 24 months of treatment due to various reasons (grade D) but due to the rebound effect, a single dose of zoledronic acid at least 6 months after denosumab discontinuation is recommended (grade D), although we believe it is good clinical practice to avoid rebound multiple vertebral fractures with bone turnover biomarkers if available (95). It is important to note that pamidronate is the second-line agent in case of contraindication to zoledronic acid or denosumab. Monitoring for adverse events or adequate calcium and vitamin D supplementation do not differ from other guidelines. We are of the belief that future RCTs should be risk-adapted, imaging- and biomarker-driven, to lead us toward precision medicine in this area. The summary of guidelines by relevant myeloma bodies are represented in **Table 4**.

**Table 4:** Guidelines for bone-modifying agents in multiple myeloma.

Guidelines	ASCO (135)	EHA-ESMO (136)	EMN (138)	IMWG [42]	NCCN (137)
Agents	Zoledronic acid, Pamidronate, Denosumab	Zoledronic acid, Denosumab	Zoledronic acid, Pamidronate, Denosumab	Zoledronic acid, Pamidronate, Denosumab	Zoledronic acid, Pamidronate, Denosumab
Indications	NDMM or RRMM	Active MM, with or without bone disease	Active MM, symptomatic or relapsed	Active MM, symptomatic or relapsed	Active MM with or without bone disease
Duration of Therapy	Up to 2 years; adjust for relapse	Up to 2 years; Continuous denosumab	12–24 months for zoledronic acid; Continuous denosumab	12–24 months for zoledronic acid; taper possible. Denosumab for 24 months, with an individualized discontinuation plan	24 months. Continuation beyond 2 years to be based on clinical judgment.
Administration	Zoledronic acid (IV, 4 mg, 15 min); Pamidronate (IV 90 mg over at least 2 hours) Denosumab (SC, 120 mg)	Per package insert	Zoledronic acid (IV, 4 mg, 15 min); Pamidronate (IV, 90 mg over at least 2 hours) Denosumab (SC, 120 mg)	Zoledronic acid (IV, 4 mg, 15 min); Pamidronate (IV, 90 mg over at least 2 hours) Denosumab (SC, 120 mg)	Per package insert
Frequency	Once monthly	Once monthly	Once monthly	Once monthly	Once every 1–3 months based on choice of agent, patient specifics, and response to treatment
Renal Impairment	Denosumab preferred for severe renal dysfunction	Denosumab for CrCl <30 mL/min	Denosumab preferred	Denosumab preferred	Denosumab preferred
Monitoring	Creatinine, calcium, vitamin D, albuminuria, monitor for ONJ	Creatinine, calcium, vitamin D, monitor for ONJ	Creatinine, calcium, vitamin D, monitor for ONJ	Creatinine, calcium, vitamin D, monitor for ONJ	Creatinine, calcium, vitamin D, monitor for ONJ
Supplementation	Calcium and vitamin D required	Calcium and vitamin D required	Calcium and vitamin D required	Calcium and vitamin D required	Calcium and vitamin D required
Dental Management	Pre-treatment dental exam; avoid invasive procedures during therapy	Dental evaluation before treatment	Dental evaluation before treatment; pause before dental procedures	Dental evaluation before treatment	Dental evaluation before treatment strongly recommended
Author Comments	- Every patient treatment plan should be individualized; - Zoledronic acid can be given once every three months [70]; - Denosumab use has the greatest risk of hypocalcemia (97); - to minimize the risk of ONJ, adequate time (8–12 weeks) should be planned from the last bone modifying agent dose to periodontal intervention/s, as well as from the time of periodontal intervention to the next dose of bone modifying agents, per clinical need. - patients who completed denosumab therapy may need maintenance denosumab every 6 months or a single dose of bisphosphonate to mitigate risk of rebound osteoporosis. - consider vertebroplasty or kyphoplasty +/- radiofrequency ablation for symptomatic vertebral compression fractures which are refractory to medical management. - attention should be placed on any potential impending fractures or structural instabilities of the spinal column, and appropriate ancillary services engaged (e.g. orthopedic or neurosurgery services)				

ASCO, American Society of Clinical Oncology; EHA-ESMO, European Hematology Association/European Society for Medical Oncology; EMN, European Myeloma Network; IMWG, International Myeloma Working Group; NCCN, National Comprehensive Cancer Network; NDMM, newly diagnosed multiple myeloma; RRMM, relapsed/refractory multiple myeloma; MM, multiple myeloma; IV, intravenous; SC, subcutaneous; ONJ, osteonecrosis of the jaw.

## Novel agents for multiple myeloma bone disease on the horizon

The treatment of MBD represents an unmet, as BPs were introduced in the early 1990s, with denosumab being only modern compound. Yet, this might change, with significant trials currently underway in this setting. To our knowledge, there is only one phase III RCT evaluating narlumosbart versus denosumab for MBD in 478 MM subjects (NCT06314698) (139). This agent inhibits the interaction between RANKL and RANK, and differs from denosumab in its monoclonal antibody structure. However, the main issue with this RCT is that only subjects of Chinese origin are being recruited, which makes it difficult to extrapolate the findings to the broader Caucasian population of Europe and the U.S., or to individuals of African origin (140). Romosozumab is a monoclonal antibody targeting sclerostin, a molecule that inhibits the Wnt pathway and promotes SREs (141). Although it has not been formally tested in MM, results in postmenopausal osteoporosis are promising, with no SREs, increased bone density, and decreased bone biomarkers of osteoclastogenesis. However, Liu et al., analyzing FAERS data, noted important safety signals such as cardiovascular disorders (ROR = 2.63) and connective tissue disorders (ROR = 2.18) (142). Furthermore, the authors could not replicate the findings of pivotal studies, with fractures and reduced bone density being common in their dataset. In conclusion, romosozumab remains a controversial compound in the treatment of osteoporosis. Further studies are essential to confirm its safety and effectiveness in the MM context. A small phase I pivotal study, including patients with osteoporosis, is currently underway, with the primary endpoint being levels of P1NP (a bone formation biomarker) and secondary endpoints related to safety (NCT05775094) (143). BHQ880 is a DKK1 neutralizing antibody shown *in vitro* to promote bone formation by stimulating osteoblasts and inhibiting proinflammatory cytokines (144). It demonstrated feasibility and safety in s phase Ib study, without dose-limiting toxicity, with a recommended dose of 10 mg/kg in 28 subjects with r-r MM (145). However, due to poor enrollment, a phase II RCT on newly diagnosed MM patients treated with the VD doublet, including those with renal insufficiency, and using time to SRE from randomization as the endpoint, is still ongoing (NCT01337752) (146). A second trial is an open-label study of BHQ880 in high-risk SMM to evaluate progression to symptomatic MM NCT01302886A (147). Activin A has become an interesting target, as it inhibits osteoblast activation and its levels are elevated in MM patients with MBD (36). A proof-of-concept phase II RCT (N = 30) in the r-r setting evaluated sotatercept in combination with MPT. Ten patients also received BPs. Subjects were divided into three cohorts receiving different dosages of a soluble fusion protein that binds activin A, while another cohort received an AII receptor (148). The primary endpoint was safety, along with assessment of skeletal bone turnover biomarkers. Reported AEs were primarily hematological, along with respiratory infections, and three patients discontinued treatment. Bone ALP levels changed significantly from baseline in patients not receiving BPs. Regarding bone density, as assessed by skeletal X-rays, improvements were observed with sotatercept, including the disappearance of one and more focal lesions compared to placebo. However, during follow-up, five SREs occurred in the ITT group, due to the fact that this compound dose does not inhibit bone resorption. Based on this trial, a phase I study is currently ongoing to assess the appropriate dose of sotatercept in combination with novel anti-MM (lenalidomide or pomalidomide) in the r-r setting, with the primary outcome being DLT (NCT01562405) (149). Another anabolic agent of interest for MBD is teriparatide, a recombinant PTH analogue, tested in a small pilot trial (N = 12), which showed increased bone density in 48% of patients (150). However, there are many concerns about this compound, primarily its potential to promote MM progression via IL-6 and hypercalcemia (151). To our knowledge, no RCT is planned for this compound in MM. The main issues with the new compounds include small sample sizes in “proof-of-concept” trials, poor enrollment in ongoing RCTs, and limited statistical power. We expect that the majority of trials on anabolic compounds will either be terminated or fail to provide the answer on how to treat MBD based on evidence-based medicine.

## Micro ribonucleic acids: their role in myeloma bone disease and possible novel targets

Micro ribonucleic acids (miRNAs) are small non-coding RNAs implicated in various diseases, such as cancer or autoimmune disorders (152–155). Their main mechanism of action is binding to mRNA and inhibiting protein translation (156). Another potential role of miRNA is facilitating cell-to-cell interactions via exosomes. In cancer, two types of miRNAs can be distinguished—oncogenic miRNAs (oncomiRs) and tumor suppressor miRNAs (tsmiRs). It is important to note that they may be used in therapy as agents (miRNA mimics) or suppressed by anti-miRs. Yet, due to off-targets effects, in phase I trial of a liposomal miRNA-34a mimic in solid cancers (N = 85) caused severe hematological and immune-related AEs, including four grade V events, and was closed prematurely—showing that we are still at the beginning of understanding how to use these agents in the clinic (157). Multiple miRNAs regulate various pathways, such as Wnt, in the pathogenesis of MM, and also carry prognostic significance (158, 159). Concerning MBD, the first *in vitro* “proof-of-concept” study showed that miR-29B downregulates osteoclast activity (160). Using a 3D bone niche model derived from MM patients and healthy controls, the authors found that five miRNAs were upregulated and one miRNA was downregulated in this model (161). Inhibition of the upregulated miRNAs did not alter the MM bone niche, while high expression of miR-199a-5p promoted osteoblastic potential via multiple pathways *in vitro*. In an *In vivo* murine model, overexpression of miR-34-a acted as an inhibitor of TGFB-Induced Factor 2 expression, decreasing MBD and MM progression (162). Circulating miRNAs also play a role in skeletal metabolism in MM, with decreased levels of miR-29c-3p being correlated with advanced MBD (163).

When comparing patients with MM and MBD to those with MM without MBD, downregulation of this miRNA was associated with bone disease (r = 0.308, AUC = 0.695, p ˂ 0.05), establishing it as a biomarker for SREs (164). Concerning osteolytic lesions, miR-29b-3p and miR-195-5p levels were associated with spine lesions. Yet, the main limitation of this study is the small sample and questionable statistical power to detect miRNA signals as biomarkers in MBD. These results should be validated on a larger sample. One of the studies in this area included 76 patients with MM and 18 patients with sMM (165). Using CD138-positive cells, the authors performed real-time qPCR to sequence the miRNAs of interest. Low expression of miR-15a-5p, miR-16-5p, and miR-222-3p was observed in MM patients with MBD, in contrast to those without bone disease, with the most prominent marker being miR-125b-5p, particularly concerning disease severity. The main value of this study is its inclusion of real-world MM patients, regardless of age, treatment, and performance status—making it a real-world evidence study. Raimondo et al. focused on extravesicular miR-129-5p and its inhibitory effects on osteoblasts (166). This extravesicular miRNA inhibited osteoblast differentiation and promoted osteoclast activity by suppressing translation of Transcription Factor SP1, one of the key regulators of osteoblastogenesis. We have presented several studies on miRNAs in MBF, but it is important to note that this field is growing rapidly. There are multiple biomarkers of MBD in MM, and additional reviews in this area are needed (36).

Despite growing interest in targeting miRNAs to treat MBD, our current clinical and translational understanding of these complex pathways and their off-target effects limits their potential use in phase I/II trials, as shown in phase I trial in solid cancers (157). Further translational and technical research is needed to determine how to deliver this agent to MM patients with adequate safety and efficacy. Until then, miRNAs will remain only prognostic markers in the area of MBD.

## Cost-effectiveness of the treatment of multiple myeloma bone disease

Formally, the cost-effectiveness of zoledronic acid has not been explored in MM, but in solid tumors, it has been shown to be the most cost-effective intravenous BP (167). Furthermore, due to the introduction of generic versions, it likely has the best cost-effective profile. The current price for annual administration through the National Health Service (NHS) is £284. Regarding pamidronate, the price per application in Australia is 55.26 Australian dollars (168). These data raise questions about the cost-effectiveness of denosumab, which costs 2, 435.11 $ per application in the U.S (169). Raje et al. performed a cost-effectiveness analysis comparing denosumab with zoledronic acid using a model that included the incidence of SREs, the cost of their management, compliance, anti-MM treatment, AEs, and outcomes in terms of PFS and OS (170). When compared to zoledronic acid, the incremental cost-effectiveness ratio (ICER) was $120, 569 per quality-adjusted life year (QALY) gained, which is below the U.S. healthcare system’s willingness-to-pay threshold of $150, 000 per intervention. However, due to the characteristics of this health care system, it is questionable how many MM patients have access to denosumab. A similar model was used in a cost-effectiveness analysis for several European countries (171). The ICER per QALY gained was as follows: €26, 294 for Austria, €17, 737 for Belgium, €6, 982 for Greece, and €27, 228 for Italy. Using gross domestic product per capita, the willingness to pay for the intervention was considered high, and denosumab was determined to be cost-effective compared to zoledronic acid. However, there are differences among various countries. The producer never submitted denosumab data for MM to the British National Institute for Health and Care Excellence, so this drug is not available through the NHS (172). In Australia, denosumab treatment is not available due to an unfavorable cost-effectiveness profile (173). While the hope is that these issues may be overcome with the expiration of the original denosumab patent and the rapid introduction of biosimilars into clinical practice, it remains to be seen if the cost of biosimilars may be meaningfully lower. Based on the USA market, this does not appear to be the cases the price of denosumab biosimilars is not meaningfully lower compared to the parent drug (**Table 3**). One of the first biosimilar, GP2411, was tested in a phase I pharmacokinetic and pharmacodynamic study on 492 healthy males, each given a subtherapeutic dose of GP2411 (35 mg), compared to the original U.S. and EU denosumab (same dose, 30 mg) randomized in a 1:1:1 ratio (174). The primary endpoint—pharmacokinetic equivalence with the originator—was met. GP2411 was tested in term of bone osteoblastogenesis markers, which were elevated in the experimental group, serving as a proof of pharmacodynamic activity. Concerning safety, no new safety signals were noted. Based on this study, the Food and Drug Administration (FDA) approved GP2411 (Wyost) as the first biosimilar of denosumab, with one of its indications being the prevention of SREs in MM and malignant solid tumors with bone metastases (175). CT_P41, a denosumab biosimilar, was compared to U.S. denosumab (60 mg dose) in a double-blind phase I study involving 154 healthy Asian males (176). Pharmacokinetic properties were equivalent to the originator, with bone markers showing improved homeostasis and adequate safety. The FDA approved CT_P41 (Osenvelt) for all indications in malignant oncology (177). Biosimilar SB16 was compared to U.S. and EU denosumab in a phase I trial on 168 subjects (178). The pharmacokinetics of SB16 were equivalent to the originators, with similar pharmacodynamics and safety. The FDA approved Xbryk, with the indication of preventing SREs in malignancies (179). It is important to note that none of these biosimilars were tested in MM. However, due to the regulatory approach for biosimilars, once a confirmatory phase III RCT is completed for one indication, extrapolation is allowed to others (180). These RCTs focused on osteoporosis and will not be explained in detail (181–183). Nonetheless, this may mark in the future a revolution in the treatment of MBD due to lower costs in the future and greater availability, positioning denosumab as the treatment of choice, yet only experienced hematologists should prescribe these medications to avoid AEs related to denosumab.

## Limitations of the review

Research on MM disease, its pathogenesis, experimental treatments, imaging, and *in vitro* and *in vivo* studies is rapidly expanding. We have tried to outline major advances in the area, and summarize the position of most relevant guideline bodies in the field. For the scope of this review article, we have limited some parts, such as bone homeostasis, bone remodeling in MM, and the role of miRNAs in SREs.

## Conclusion

MBD is a cause of morbidity and mortality in patients suffering from MM (6). BPs were introduced in the 1990s, with denosumab being the only relatively new agent for the treatment and prevention of SREs in this setting (47, 49, 51, 90). It is important to note that while all BPs are equally potent, zoledronic acid is preferred due to patient convenience (57) as well as significant price difference in all markets. Furthermore, BPs in combination with anti-MM therapy appear to impact the disease course, leading to improved PFS and OS, although the actual clinical benefit remains questionable (5). However, we must be aware that these agents carry significant risk of AEs, most notably MRONJ, and various renal disorders (73, 76). Nevertheless, where price does not represent a barrier, denosumab may be generally preferred in patients with renal insufficiency, with monitoring and prevention of AEs. Yet, we are in an era of personalized, precision medicine. Imaging studies and bone turnover biomarkers, such as miRNAs, combined with advancing technologies may guide a risk-adapted approach to the treatment of MBD (184). However, RCTs in this area face challenges including limited regional availability, while price of biosimilar agents remains comparably high, making monoclonal agents a prohibitive factor, even in many developed nations.

## References

[1] Medeiros LJ Chadburn A Natkunam Y Naresh KN WHO 5th Edition Classification Project . Fifth edition of the World Health Classification of Tumors of the Hematopoietic and Lymphoid Tissues: B-cell neoplasms. Mod Pathol. (2024) 37:100441. doi: 10.1016/j.modpat.2024.100441. PMID: 38309432

[2] Campo E Jaffe ES Cook JR Quintanilla-Martinez L Swerdlow SH Anderson KC . The international consensus classification of mature lymphoid neoplasms: a report from the clinical advisory committee. Blood. (2022) 140:1229–53. doi: 10.1182/blood.2022015851. PMID: 35653592 PMC9479027

[3] Surveillance, Epidemiology and End Results Program . Cancer stat facts: myeloma (2025). Available online at: https://seer.cancer.gov/statfacts/html/mulmy.html (Accessed October 1, 2025).

[4] Padala SA Barsouk A Barsouk A Rawla P Vakiti A Kolhe R . Epidemiology, staging, and management of multiple myeloma. Med Sci (Basel). (2021) 9:3. doi: 10.3390/medsci9010003. PMID: 33498356 PMC7838784

[5] Kyle RA Gertz MA Witzig TE Lust JA Lacy MQ Dispenzieri A . Review of 1027 patients with newly diagnosed multiple myeloma. Mayo Clin Proc. (2003) 78:21–33. doi: 10.4065/78.1.21. PMID: 12528874

[6] Bernstein ZS Kim EB Raje N . Bone disease in multiple myeloma: biologic and clinical implications. Cells. (2022) 11:2308. doi: 10.3390/cells11152308. PMID: 35954151 PMC9367243

[7] Terpos E Berenson J Cook RJ Lipton A Coleman RE . Prognostic variables for survival and skeletal complications in patients with multiple myeloma osteolytic bone disease. Leukemia. (2010) 24:1043–9. doi: 10.1038/leu.2010.62. PMID: 20376081

[8] Edwards CM Zhuang J Mundy GR . The pathogenesis of the bone disease of multiple myeloma. Bone. (2008) 42:1007–13. doi: 10.1016/j.bone.2008.01.027. PMID: 18406675 PMC2474770

[9] Moreau P Attal M Caillot D Macro M Karlin L Garderet L . Prospective evaluation of magnetic resonance imaging and [18F]fluorodeoxyglucose positron emission tomography-computed tomography at diagnosis and before maintenance therapy in symptomatic patients with multiple myeloma included in the IFM/DFCI 2009 trial: Results of the IMAJEM study. J Clin Oncol. (2017) 35:2911–8. doi: 10.1200/JCO.2017.72.2975. PMID: 28686535 PMC5578392

[10] Hillengass J Usmani S Rajkumar SV Durie B Mateos MV Lonial S . International myeloma working group consensus recommendations on imaging in monoclonal plasma cell disorders. Lancet Oncol. (2019) 20:e302–12. doi: 10.1016/S1470-2045(19)30309-2. PMID: 31162104

[11] Wu Z Li W Jiang K Lin Z Qian C Wu M . Regulation of bone homeostasis: signaling pathways and therapeutic targets. MedComm (2020). (2024) 5:e657. doi: 10.1002/mco2.657. PMID: 39049966 PMC11266958

[12] Seeman E . Bone modeling and remodeling. Crit Rev Eukaryot Gene Expr. (2009) 19:219–33. doi: 10.1615/critreveukargeneexpr.v19.i3.40. PMID: 19883366

[13] Zhu S Chen W Masson A Li YP . Cell signaling and transcriptional regulation of osteoblast lineage commitment, differentiation, bone formation, and homeostasis. Cell Discov. (2024) 10:71. doi: 10.1038/s41421-024-00689-6. PMID: 38956429 PMC11219878

[14] Capulli M Paone R Rucci N . Osteoblast and osteocyte: games without frontiers. Arch Biochem Biophys. (2014) 561:3–12. doi: 10.1016/j.abb.2014.05.003. PMID: 24832390

[15] Teitelbaum SL Ross FP . Genetic regulation of osteoclast development and function. Nat Rev Genet. (2003) 4:638–49. doi: 10.1038/nrg1122. PMID: 12897775

[16] Wada T Nakashima T Hiroshi N Penninger JM . RANKL-RANK signaling in osteoclastogenesis and bone disease. Trends Mol Med. (2006) 12:17–25. doi: 10.1016/j.molmed.2005.11.007. PMID: 16356770

[17] Anderson DM Maraskovsky E Billingsley WL Dougall WC Tometsko ME Roux ER . A homologue of the TNF receptor and its ligand enhance T-cell growth and dendritic-cell function. Nature. (1997) 390:175–9. doi: 10.1038/36593. PMID: 9367155

[18] Boyce BF Xing L . Functions of RANKL/RANK/OPG in bone modeling and remodeling. Arch Biochem Biophys. (2008) 473:139–46. doi: 10.1016/j.abb.2008.03.018. PMID: 18395508 PMC2413418

[19] Maciel GBM Maciel RM Danesi CC . Bone cells and their role in physiological remodeling. Mol Biol Rep. (2023) 50:2857–63. doi: 10.1007/s11033-022-08190-7. PMID: 36609750

[20] Park-Min KH Mun SH Bockman R McDonald MM . New horizons: translational aspects of osteomorphs. J Clin Endocrinol Metab. (2024) 109:e1373–8. doi: 10.1210/clinem/dgad711. PMID: 38060842 PMC11031245

[21] McDonald MM Khoo WH Ng PY Xiao Y Zamerli J Thatcher P . Osteoclasts recycle via osteomorphs during RANKL-stimulated bone resorption. Cell. (2021) 184:1330–1347.e13. doi: 10.1016/j.cell.2021.02.002. PMID: 33636130 PMC7938889

[22] Simonet WS Lacey DL Dunstan CR Kelley M Chang MS Lüthy R . Osteoprotegerin: a novel secreted protein involved in the regulation of bone density. Cell. (1997) 89:309–19. doi: 10.1016/s0092-8674(00)80209-3. PMID: 9108485

[23] Yao Z Getting SJ Locke IC . Regulation of TNF-induced osteoclast differentiation. Cells. (2021) 11:132. doi: 10.3390/cells11010132. PMID: 35011694 PMC8750957

[24] Leszczyńska D Szatko A Toboła A Karoń K Misiorowski W Glinicki P . Novel aspects of biochemical assessment of bone remodeling and mineralization. Front Endocrinol (Lausanne). (2025) 16:1702413. doi: 10.3389/fendo.2025.1702413. PMID: 41367909 PMC12682636

[25] Terpos E Ntanasis-Stathopoulos I Gavriatopoulou M Dimopoulos MA . Pathogenesis of bone disease in multiple myeloma: from bench to bedside. Blood Cancer J. (2018) 8:7. doi: 10.1038/s41408-017-0037-4. PMID: 29330358 PMC5802524

[26] Delgado-Calle J Anderson J Cregor MD Hiasa M Chirgwin JM Carlesso N . Bidirectional Notch signaling and osteocyte-derived factors in the bone marrow microenvironment promote tumor cell proliferation and bone destruction in multiple myeloma. Cancer Res. (2016) 76:1089–100. doi: 10.1158/0008-5472.CAN-15-1703. PMID: 26833121 PMC4775415

[27] Giuliani N Colla S Sala R Moroni M Lazzaretti M La Monica S . Human myeloma cells stimulate the receptor activator of nuclear factor-kappa B ligand (RANKL) in T lymphocytes: a potential role in multiple myeloma bone disease. Blood. (2002) 100:4615–21. doi: 10.1182/blood-2002-04-1121. PMID: 12393684

[28] Terpos E Szydlo R Apperley JF Hatjiharissi E Politou M Meletis J . Soluble receptor activator of nuclear factor kappaB ligand-osteoprotegerin ratio predicts survival in multiple myeloma: proposal for a novel prognostic index. Blood. (2003) 102:1064–9. doi: 10.1182/blood-2003-02-0380. PMID: 12689925

[29] Palma BD Guasco D Pedrazzoni M Bolzoni M Accardi F Costa F . Osteolytic lesions, cytogenetic features and bone marrow levels of cytokines and chemokines in multiple myeloma patients: Role of chemokine (C-C motif) ligand 20. Leukemia. (2016) 30:409–16. doi: 10.1038/leu.2015.259. PMID: 26419509

[30] Sugatani T Alvarez UM Hruska KA . Activin A stimulates IkappaB-alpha/NFkappaB and RANK expression for osteoclast differentiation, but not AKT survival pathway in osteoclast precursors. J Cell Biochem. (2003) 90:59–67. doi: 10.1002/jcb.10613. PMID: 12938156

[31] Lee JW Chung HY Ehrlich LA Jelinek DF Callander NS Roodman GD . IL-3 expression by myeloma cells increases both osteoclast formation and growth of myeloma cells. Blood. (2004) 103:2308–15. doi: 10.1182/blood-2003-06-1992. PMID: 14615378

[32] Lam J Takeshita S Barker JE Kanagawa O Ross FP Teitelbaum SL . TNF-alpha induces osteoclastogenesis by direct stimulation of macrophages exposed to permissive levels of RANK ligand. J Clin Invest. (2000) 106:1481–8. doi: 10.1172/JCI11176. PMID: 11120755 PMC387259

[33] Hiasa M Harada T Tanaka E Abe M . Pathogenesis and treatment of multiple myeloma bone disease. Jpn Dent Sci Rev. (2021) 57:164–73. doi: 10.1016/j.jdsr.2021.08.006. PMID: 34611468 PMC8477206

[34] Fowler JA Mundy GR Lwin ST Edwards CM . Bone marrow stromal cells create a permissive microenvironment for myeloma development: a new stromal role for Wnt inhibitor Dkk1. Cancer Res. (2012) 72:2183–9. doi: 10.1158/0008-5472.CAN-11-2067. PMID: 22374979 PMC3775476

[35] Ehrlich LA Chung HY Ghobrial I Choi SJ Morandi F Colla S . IL-3 is a potential inhibitor of osteoblast differentiation in multiple myeloma. Blood. (2005) 106:1407–14. doi: 10.1182/blood-2005-03-1080. PMID: 15878977

[36] Vallet S Mukherjee S Vaghela N Hideshima T Fulciniti M Pozzi S . Activin A promotes multiple myeloma-induced osteolysis and is a promising target for myeloma bone disease. Proc Natl Acad Sci USA. (2010) 107:5124–9. doi: 10.1073/pnas.0911929107. PMID: 20194748 PMC2841922

[37] Terpos E Kastritis E Christoulas D Gkotzamanidou M Eleutherakis-Papaiakovou E Kanellias N . Circulating activin-A is elevated in patients with advanced multiple myeloma and correlates with extensive bone involvement and inferior survival; no alterations post-lenalidomide and dexamethasone therapy. Ann Oncol. (2012) 23:2681–6. doi: 10.1093/annonc/mds068. PMID: 22492699

[38] Goel U Usmani S Kumar S . Current approaches to management of newly diagnosed multiple myeloma. Am J Hematol. (2022) 97:S3–S25. doi: 10.1002/ajh.26512. PMID: 35234302

[39] Zhang SC Ballas LK . Radiation for multiple myeloma in the era of novel agents: indications, safety, and dose selection. Semin Radiat Oncol. (2025) 35:87–98. doi: 10.1016/j.semradonc.2024.10.004. PMID: 39672645

[40] Zehri AH Calafiore RL Peterson KA Kittel CA Osei JA Wilson JL . Surgical management of spinal multiple myeloma: insights from the National Inpatient Sample database. J Spine Surg. (2024) 10:428–37. doi: 10.21037/jss-24-54. PMID: 39399072 PMC11467281

[41] Hussain M Khan F Al Hadidi S . The use of bone-modifying agents in multiple myeloma. Blood Rev. (2023) 57:100999. doi: 10.1016/j.blre.2022.10099. PMID: 36050125

[42] Terpos E Zamagni E Lentzsch S Drake MT García-Sanz R Abildgaard N . Treatment of multiple myeloma-related bone disease: recommendations from the Bone Working Group of the International Myeloma Working Group. Lancet Oncol. (2021) 22:e119–30. doi: 10.1016/S1470-2045(20)30559-3. PMID: 33545067

[43] Terpos E Ntanasis-Stathopoulos I . Controversies in the use of new bone-modifying therapies in multiple myeloma. Br J Haematol. (2021) 193:1034–43. doi: 10.1111/bjh.17256. PMID: 33249579

[44] McCloskey E Paterson AH Powles T Kanis JA . Clodronate. Bone. (2021) 143:115715. doi: 10.1016/j.bone.2020.115715. PMID: 33127577

[45] Belch AR Bergsagel DE Wilson K O'Reilly S Wilson J Sutton D . Effect of daily etidronate on the osteolysis of multiple myeloma. J Clin Oncol. (1991) 9:1397–402. doi: 10.1200/JCO.1991.9.8.1397. PMID: 1712835

[46] McCloskey EV MacLennan IC Drayson MT Chapman C Dunn J Kanis JA . A randomized trial of the effect of clodronate on skeletal morbidity in multiple myeloma. MRC Working Party on Leukaemia in Adults. Br J Haematol. (1998) 100:317–25. doi: 10.1046/j.1365-2141.1998.00567.x. PMID: 9488619

[47] Lahtinen R Laakso M Palva I Virkkunen P Elomaa I . Randomised, placebo-controlled multicentre trial of clodronate in multiple myeloma. Finnish Leukaemia Group. Lancet. (1992) 340:1049–52. doi: 10.1016/0140-6736(92)93075-x. PMID: 1357451

[48] Frediani B Baraldi E Cremonesi G . Effect of clodronate treatment on risk of fracture: a systematic review and meta-analysis. Calcif Tissue Int. (2014) 95:295–307. doi: 10.1007/s00223-014-9903-2. PMID: 25113241

[49] Berenson JR Lichtenstein A Porter L Dimopoulos MA Bordoni R George S . Efficacy of pamidronate in reducing skeletal events in patients with advanced multiple myeloma. Myeloma Aredia Study Group. N Engl J Med. (1996) 334:488–93. doi: 10.1056/NEJM199602223340802. PMID: 8559201

[50] Berenson JR Lichtenstein A Porter L Dimopoulos MA Bordoni R George S . Long-term pamidronate treatment of advanced multiple myeloma patients reduces skeletal events. Myeloma Aredia Study Group. J Clin Oncol. (1998) 16:593–602. doi: 10.1200/JCO.1998.16.2.593. PMID: 9469347

[51] Terpos E Ntanasis-Stathopoulos M Morgan GJ Child JA Gregory WM Szubert AJ . Effects of zoledronic acid versus clodronic acid on skeletal morbidity in patients with newly diagnosed multiple myeloma (MRC Myeloma IX): secondary outcomes from a randomised controlled trial. Lancet Oncol. (2011) 12:743–52. doi: 10.1016/S1470-2045(11)70157-7. PMID: 21771568 PMC3148431

[52] Djulbegovic B Wheatley K Ross J Clark O Bos G Goldschmidt H . Bisphosphonates in multiple myeloma. Cochrane Database Syst Rev. (2001) 4:CD003188. doi: 10.1002/14651858.CD003188. PMID: 11687178

[53] Dunford JE Thompson K Coxon FP Luckman SP Hahn FM Poulter CD . Structure-activity relationships for inhibition of farnesyl diphosphate synthase *in vitro* and inhibition of bone resorption *in vivo* by nitrogen-containing bisphosphonates. J Pharmacol Exp Ther. (2001) 296:235–42. doi: 10.1016/s0022-3565(24)38786-5. PMID: 11160603

[54] Morgan GJ Davies FE Gregory WM Bell SE Szubert AJ Cook G . Long-term follow-up of MRC Myeloma IX trial: Survival outcomes with bisphosphonate and thalidomide treatment. Clin Cancer Res. (2013) 19:6030–8. doi: 10.1158/1078-0432.CCR-12-3211. PMID: 23995858

[55] Sanfilippo KM Gage B Luo S Weilbaecher K Tomasson M Vij R . Comparative effectiveness on survival of zoledronic acid versus pamidronate in multiple myeloma. Leuk Lymphoma. (2015) 56:615–21. doi: 10.3109/10428194.2014.924117. PMID: 24844358 PMC4399231

[56] Mhaskar R Redzepovic J Wheatley K Clark OA Miladinovic B Glasmacher A . Bisphosphonates in multiple myeloma: a network meta-analysis. Cochrane Database Syst Rev. (2012), (5):CD003188. doi: 10.1002/14651858.CD003188.pub3. PMID: 22592688

[57] Bastounis A Langley T Davis S Paskins Z Gittoes N Leonardi-Bee J . Comparing medication adherence in patients receiving bisphosphonates for preventing fragility fractures: a comprehensive systematic review and network meta-analysis. Osteoporos Int. (2022) 33:1223–33. doi: 10.1007/s00198-022-06350-w. PMID: 35188591 PMC9106630

[58] Trotter TN Fok M Gibson JT Peker D Javed A Yang Y . Osteocyte Apoptosis Attracts Myeloma Cells to Bone and Supports Progression through Regulation of the Bone Marrow Microenvironment (2016). Available online at: https://www.sciencedirect.com/science/article/pii/S0006497119304859.

[59] Plotkin LI Weinstein RS Parfitt AM Roberson PK Manolagas SC Bellido T . Prevention of osteocyte and osteoblast apoptosis by bisphosphonates and calcitonin. J Clin Invest. (1999) 104:1363–74. doi: 10.1172/JCI6800. PMID: 10562298 PMC409837

[60] Mori Y Shimizu N Dallas M Niewolna M Story B Williams PJ . Anti-alpha4 integrin antibody suppresses the development of multiple myeloma and associated osteoclastic osteolysis. Blood. (2004) 104:2149–54. doi: 10.1182/blood-2004-01-0236. PMID: 15138161

[61] Vanderkerken K Medicherla S Coulton L De Raeve H Willems A Lawson M . Inhibition of p38alpha mitogen-activated protein kinase prevents the development of osteolytic bone disease, reduces tumor burden, and increases survival in murine models of multiple myeloma. Cancer Res. (2007) 67:4572–7. doi: 10.1158/0008-5472.CAN-06-4361. PMID: 17495322

[62] Terpos E Dimopoulos MA . Interaction between the skeletal and immune systems in cancer: mechanisms and clinical implications. Cancer Immunol Immunother. (2011) 60:305–17. doi: 10.1007/s00262-011-0974-x. PMID: 21243489 PMC11028766

[63] McCloskey EV Dunn JA Kanis JA MacLennan IC Drayson MT . Long-term follow-up of a prospective, double-blind, placebo-controlled randomized trial of clodronate in multiple myeloma. Br J Haematol. (2001) 113:1035–43. doi: 10.1046/j.1365-2141.2001.02851.x. PMID: 11442499

[64] Mhaskar R Kumar A Miladinovic B Djulbegovic B . Bisphosphonates in multiple myeloma: an updated network meta-analysis. Cochrane Database Syst Rev. (2017) 12:CD003188. doi: 10.1002/14651858.CD003188.pub4. PMID: 29253322 PMC6486151

[65] Campagnaro E Reimers MA Qin A Alva AS Schneider BJ Van Poznak CH . Use of bone-modifying agents in myeloma and bone metastases: how recent dosing interval studies have affected our practice. J Oncol Pract. (2018) 14:457–64. doi: 10.1200/JOP.18.00236. PMID: 30096277

[66] Gimsing P Carlson K Turesson I Fayers P Waage A Vangsted A . Effect of pamidronate 30 mg versus 90 mg on physical function in patients with newly diagnosed multiple myeloma (Nordic Myeloma Study Group): a double-blind, randomised controlled trial. Lancet Oncol. (2010) 11:973–82. doi: 10.1016/S1470-2045(10)70198-4. PMID: 20863761

[67] Aaronson NK Ahmedzai S Bergman B Bullinger M Cull A Duez NJ . The European Organization for Research and Treatment of Cancer QLQ-C30: a quality-of-life instrument for use in international clinical trials in oncology. J Natl Cancer Inst. (1993) 85:365–76. doi: 10.1093/jnci/85.5.365. PMID: 8433390

[68] Himelstein AL Foster JC Khatcheressian JL Roberts JD Seisler DK Novotny PJ . Effect of longer-interval vs standard dosing of zoledronic acid on skeletal events in patients with bone metastases: A randomized clinical trial. JAMA. (2017) 317:48–58. doi: 10.1001/jama.2016.19425. PMID: 28030702 PMC5321662

[69] Van den Wyngaert T Delforge M Doyen C Duck L Wouters K Delabaye I . Prospective observational study of treatment pattern, effectiveness and safety of zoledronic acid therapy beyond 24 months in patients with multiple myeloma or bone metastases from solid tumors. Support Care Cancer. (2013) 21:3483–90. doi: 10.1007/s00520-013-1934-0. PMID: 23955094

[70] Avilès A Nambo MJ Huerta-Guzmàn J Cleto S Neri N . Prolonged use of zoledronic acid (4 years) did not improve outcome in multiple myeloma patients. Clin Lymphoma Myeloma Leuk. (2017) 17:207–10. doi: 10.1016/j.clml.2017.02.007. PMID: 28284745

[71] Lund T Gundesen MT Juul Vangsted A Helleberg C Haukås E Silkjær T . In multiple myeloma, monthly treatment with zoledronic acid beyond two years offers sustained protection against progressive bone disease. Blood Cancer J. (2024) 14:65. doi: 10.1038/s41408-024-01046-2. PMID: 38622134 PMC11018794

[72] Raje N Vescio R Montgomery CW Badros A Munshi N Orlowski R . Bone marker-directed dosing of zoledronic acid for the prevention of skeletal complications in patients with multiple myeloma: results of the Z-MARK study. Clin Cancer Res. (2016) 22:1378–84. doi: 10.1158/1078-0432.CCR-15-1864. PMID: 26644410

[73] Wang Z Su X Shi D Wei L . Evaluate the renal system damage caused by zoledronic acid: a comprehensive analysis of adverse events from FAERS. BMC Cancer. (2024) 24:1520. doi: 10.1186/s12885-024-13284-5. PMID: 39695477 PMC11657706

[74] Dimopoulos MA Merlini G Bridoux F Leung N Mikhael J Harrison SJ . Management of multiple myeloma-related renal impairment: recommendations from the International Myeloma Working Group. Lancet Oncol. (2023) 24:e293–311. doi: 10.1016/S1470-2045(23)00223-1. PMID: 37414019

[75] Ho PJ Moore EM McQuilten ZK Wellard C Bergin K Augustson B . Renal impairment at diagnosis in myeloma: patient characteristics, treatment, and impact on outcomes. Results from the Australia and New Zealand myeloma and related diseases registry. Clin Lymphoma Myeloma Leuk. (2019) 19:e415–24. doi: 10.1016/j.clml.2019.05.010. PMID: 31208889

[76] Olofsson R Korytowska M Almhöjd U Almståhl A Cevik-Aras H . Oral health, dental treatment, and medication related osteonecrosis of the jaw in multiple myeloma - a longitudinal cohort study. BMC Oral Health. (2024) 24:184. doi: 10.1186/s12903-024-03943-1. PMID: 38317122 PMC10840162

[77] Coppini M Arduini S Mauceri R Perez IAT Campisi G . Low awareness of medication-related osteonecrosis of the jaw among dentists: a systematic review with a medical and bioethical perspective. BMC Med Ethics. (2026) 27:19. doi: 10.1186/s12910-025-01361-8. PMID: 41540420 PMC12892618

[78] Black DM Geiger EJ Eastell R Vittinghoff E Li BH Ryan DS . Atypical femur fracture risk versus fragility fracture prevention with bisphosphonates. N Engl J Med. (2020) 383:743–53. doi: 10.1056/NEJMoa1916525. PMID: 32813950 PMC9632334

[79] Gougeon A Granal M Massy E Gueyffier F Lega JC Lajoinie A . How publication bias overestimates the risk of atypical femoral fracture and osteonecrosis of the jaw associated with bisphosphonate use: A meta-epidemiological study. Joint Bone Spine. (2025) 92:105871. doi: 10.1016/j.jbspin.2025.105871. PMID: 40015362

[80] Bejhed RS Kharazmi M Hallberg P . Identification of risk factors for bisphosphonate-associated atypical femoral fractures and osteonecrosis of the jaw in a pharmacovigilance database. Ann Pharmacother. (2016) 50:616–24. doi: 10.1177/1060028016649368. PMID: 27179251

[81] Chennuru S Koduri J Baumann MA . Risk factors for symptomatic hypocalcaemia complicating treatment with zoledronic acid. Intern Med J. (2008) 38:635–7. doi: 10.1111/j.1445-5994.2007.01580.x. PMID: 18284458

[82] Duval M Bach-Ngohou K Masson D Guimard C Le Conte P Trewick D . Is severe hypocalcemia immediately life threatening? Endocr Connect. (2018) 7:1067–74. doi: 10.1530/EC-18-0267. PMID: 30311756 PMC6198192

[83] Berruti A Tucci M Generali D Mosca A Ardine M Vana F . Management of the side-effects of intravenous bisphosphonates: targeting the serum parathyroid hormone elevation. Ann Oncol. (2006) 17:1854–5. doi: 10.1093/annonc/mdl181. PMID: 16936183

[84] Hanley DA Adachi JD Bell A Brown V . Denosumab: mechanism of action and clinical outcomes. Int J Clin Pract. (2012) 66:1139–46. doi: 10.1111/ijcp.12022. PMID: 22967310 PMC3549483

[85] Marathe A Peterson MC Mager DE . Integrated cellular bone homeostasis model for denosumab pharmacodynamics in multiple myeloma patients. J Pharmacol Exp Ther. (2008) 326:555–62. doi: 10.1124/jpet.108.137703. PMID: 18460643 PMC2574593

[86] Cummings SR San Martin J McClung MR Siris ES Eastell R Reid IR . Denosumab for prevention of fractures in postmenopausal women with osteoporosis. N Engl J Med. (2009) 361:756–65. doi: 10.1056/NEJMoa0809493. PMID: 19671655

[87] Henry DH Costa L Goldwasser F Hirsh V Hungria V Prausova J . Randomized, double-blind study of denosumab versus zoledronic acid in the treatment of bone metastases in patients with advanced cancer (excluding breast and prostate cancer) or multiple myeloma. J Clin Oncol. (2011) 29:1125–32. doi: 10.1200/JCO.2010.31.3304. PMID: 21343556

[88] Vadhan-Raj S von Moos R Fallowfield LJ Patrick DL Goldwasser F Cleeland CS . Clinical benefit in patients with metastatic bone disease: results of a phase 3 study of denosumab versus zoledronic acid. Ann Oncol. (2012) 23:3045–51. doi: 10.1093/annonc/mds175. PMID: 22851406

[89] Raje N Vadhan-Raj S Willenbacher W Terpos E Hungria V Spencer A . Evaluating results from the multiple myeloma patient subset treated with denosumab or zoledronic acid in a randomized phase 3 trial. Blood Cancer J. (2016) 6:e378. doi: 10.1038/bcj.2015.96. PMID: 26745852 PMC4742634

[90] Raje N Terpos E Willenbacher W Shimizu K García-Sanz R Durie B . Denosumab versus zoledronic acid in bone disease treatment of newly diagnosed multiple myeloma: an international, double-blind, double-dummy, randomised, controlled, phase 3 study. Lancet Oncol. (2018) 19:370–81. doi: 10.1016/S1470-2045(18)30072-X. PMID: 29429912

[91] Terpos E Raje N Croucher P Garcia-Sanz R Leleu X Pasteiner W . Denosumab compared with zoledronic acid on PFS in multiple myeloma: exploratory results of an international phase 3 study. Blood Adv. (2021) 5:725–36. doi: 10.1182/bloodadvances.2020002378. PMID: 33560384 PMC7876889

[92] Kumar S Wang M Kim AS Center JR McDonald MM Girgis CM . Denosumab discontinuation in the clinic: implications of rebound bone turnover and emerging strategies to prevent bone loss and fractures. J Bone Miner Res. (2025) 40:1017–34. doi: 10.1093/jbmr/zjaf037. PMID: 40057981 PMC12406127

[93] Bone HG Wagman RB Brandi ML Brown JP Chapurlat R Cummings SR . 10 years of denosumab treatment in postmenopausal women with osteoporosis: results from the phase 3 randomised FREEDOM trial and open-label extension. Lancet Diabetes Endocrinol. (2017) 5:513–23. doi: 10.1016/S2213-8587(17)30138-9. PMID: 28546097

[94] Cummings SR Ferrari S Eastell R Gilchrist N Jensen JB McClung M . Vertebral fractures after discontinuation of denosumab: A post hoc analysis of the randomized placebo-controlled FREEDOM trial and its extension. J Bone Miner Res. (2018) 33:190–8. doi: 10.1002/jbmr.3337. PMID: 29105841

[95] Tsourdi E Zillikens MC Meier C Body JJ Gonzalez Rodriguez E Anastasilakis AD . Fracture risk and management of discontinuation of denosumab therapy: a systematic review and position statement by ECTS. J Clin Endocrinol Metab. (2021) 106(1):264–281. doi: 10.1210/clinem/dgaa756. PMID: 33103722

[96] Dimopoulos MA Terpos E Chanan-Khan A Leung N Ludwig H Jagannath S . Renal impairment in patients with multiple myeloma: a consensus statement on behalf of the International Myeloma Working Group. J Clin Oncol. (2010) 28:4976–84. doi: 10.1200/JCO.2010.30.8791. PMID: 20956629

[97] Lee JH Heo SJ Kim KM Lee JE . Trends in serum phosphate, calcium, and parathyroid hormone levels according to renal function following denosumab. Osteoporos Int. (2025) 36:1661–9. doi: 10.1007/s00198-025-07601-2. PMID: 40650741

[98] Bird ST Smith ER Gelperin K Jung TH Thompson A Kambhampati R . Hypocalcemia with denosumab among older female dialysis-dependent patients. JAMA. (2024) 331:491–9. doi: 10.1001/jama.2023.28239. PMID: 38241060 PMC10799290

[99] Bird ST Gelperin K Smith ER Jung TH Lyu H Thompson A . The effect of denosumab on risk for emergently treated hypocalcemia by stage of chronic kidney disease: a target trial emulation. Ann Intern Med. (2025) 178:29–38. doi: 10.7326/M24-0013. PMID: 39556837

[100] Bai H Wang J Han X Li H Nie X Wang G . Risk factors for denosumab-related osteonecrosis of the jaw: a pharmacovigilance study using the U.S. FDA adverse event reporting system database. Front Endocrinol (Lausanne). (2025) 16:1586626. doi: 10.3389/fendo.2025.1586626. PMID: 41488140 PMC12757244

[101] van de Laarschot DM McKenna MJ Abrahamsen B Langdahl B Cohen-Solal M Guañabens N . Medical management of patients after atypical femur fractures: a systematic review and recommendations from the European Calcified Tissue Society. J Clin Endocrinol Metab. (2020) 105:1682–99. doi: 10.1210/clinem/dgz295. PMID: 31867670 PMC7121199

[102] Durie BG . The role of anatomic and functional staging in myeloma: description of Durie/Salmon plus staging system. Eur J Cancer. (2006) 42(11):1539–43. doi: 10.1016/j.ejca.2005.11.037. PMID: 16777405

[103] Liu Y Huang W Yang Y Cai W Sun Z . Recent advances in imaging and artificial intelligence (AI) for quantitative assessment of multiple myeloma. Am J Nucl Med Mol Imaging. (2024) 14:208–29. doi: 10.62347/NLLV9295. PMID: 39309415 PMC11411189

[104] Hillengass J Fechtner K Weber MA Bäuerle T Ayyaz S Heiss C . Prognostic significance of focal lesions in whole-body magnetic resonance imaging in patients with asymptomatic multiple myeloma. J Clin Oncol. (2010) 28:1606–10. doi: 10.1200/JCO.2009.25.5356. PMID: 20177023

[105] Zamagni E Patriarca F Nanni C Zannetti B Englaro E Pezzi A . Prognostic relevance of 18-F FDG PET/CT in newly diagnosed multiple myeloma patients treated with up-front autologous transplantation. Blood. (2011) 118:5989–95. doi: 10.1182/blood-2011-06-361386. PMID: 21900189

[106] Cho HJ Baek DW Kim JH Lee J Chung YK Jung SH . Favorable long-term outcomes with autologous stem cell transplantation for high-risk multiple myeloma patients with a positive result on 18F-FDG PET/CT at baseline. Clin Lymphoma Myeloma Leuk. (2022) 22:113–20. doi: 10.1016/j.clml.2021.08.012. PMID: 34598908

[107] Palumbo A Avet-Loiseau H Oliva S Lokhorst HM Goldschmidt H Rosinol L . Revised international staging system for multiple myeloma: a report from international myeloma working group. J Clin Oncol. (2015) 33:2863–9. doi: 10.1200/JCO.2015.61.2267. PMID: 26240224 PMC4846284

[108] Lee H Hyun SH Cho YS Moon SH Choi JY Kim K . Semi-quantitative FDG parameters predict survival in multiple myeloma patients without autologous stem cell transplantation. Cancer Imaging. (2023) 23:104. doi: 10.1186/s40644-023-00625-z. PMID: 37891633 PMC10612180

[109] Lecouvet FE Boyadzhiev D Collette L Moon SH Choi JY Kim K . MRI versus 18F-FDG-PET/CT for detecting bone marrow involvement in multiple myeloma: diagnostic performance and clinical relevance. Eur Radiol. (2020) 30:1927–37. doi: 10.1007/s00330-019-06469-1. PMID: 31844960

[110] Li Y Yin P Liu Y Chen L Sun C Wang S . Radiomics models based on magnetic resonance imaging for prediction of the response to bortezomib-based therapy in patients with multiple myeloma. BioMed Res Int. (2022) 2022:6911246. doi: 10.1155/2022/6911246. PMID: 36105939 PMC9467708

[111] Sun M Wang L Wang C Wang W Lin L Ren C . Quantitative analysis of whole-body MRI for accessing the degree of diffuse infiltration patterns and identifying high risk cases of newly diagnosed multiple myeloma. J Magn Reson Imaging. (2024) 59:2035–45. doi: 10.1002/jmri.28962. PMID: 37675995

[112] Rasche L Angtuaco EJ Alpe TL Gershner GH McDonald J Samant RS . The presence of large focal lesions is a strong independent prognostic factor in multiple myeloma. Blood. (2018) 132:59–66. doi: 10.1182/blood-2018-04-842880. PMID: 29784643 PMC6034645

[113] Hongming H Jian H . Bortezomib inhibits maturation and function of osteoclasts from PBMCs of patients with multiple myeloma by downregulating TRAF6. Leuk Res. (2009) 33:115–22. doi: 10.1016/j.leukres.2008.07.028. PMID: 18778854

[114] Lee SH Yun SJ Kim DH Jo HH Song JG Park YS . Diagnostic usefulness of low-dose lumbar multi-detector CT with iterative reconstruction in trauma patients: a comparison with standard-dose CT. Br J Radiol. (2017) 90:20170181. doi: 10.1259/bjr.20170181. PMID: 28707527 PMC5858793

[115] Quinn B Dauer Z Pandit-Taskar N Schoder H Dauer LT . Radiation dosimetry of 18F-FDG PET/CT: incorporating exam-specific parameters in dose estimates. BMC Med Imaging. (2016) 16:41. doi: 10.1186/s12880-016-0143-y. PMID: 27317478 PMC4912712

[116] American Cancer Society . Cancer facts and figures 2025. Available online at: https://www.cancer.org/content/dam/cancer-org/research/cancer-facts-and-statistics/annual-cancer-facts-and-figures/2025/2025-cancer-facts-and-figures-acs.pdf (Accessed October 1, 2025).

[117] Musto P Anderson KC Attal M Richardson PG Badros A Hou J . Second primary Malignancies in multiple myeloma: an overview and IMWG consensus. Ann Oncol. (2017) 28:228–45. doi: 10.1093/annonc/mdw606. PMID: 27864218

[118] Mohty M Malard F Mohty B Savani B Moreau P Terpos E . The effects of bortezomib on bone disease in patients with multiple myeloma. Cancer. (2014) 120:618–23. doi: 10.1002/cncr.28481. PMID: 24249482

[119] Richardson PG Sonneveld P Schuster M Irwin D Stadtmauer E Facon T . Extended follow-up of a phase 3 trial in relapsed multiple myeloma: final time-to-event results of the APEX trial. Blood. (2007) 110:3557–60. doi: 10.1182/blood-2006-08-036947. PMID: 17690257

[120] Zangari M Esseltine D Lee CK Barlogie B Elice F Burns MJ . Response to bortezomib is associated to osteoblastic activation in patients with multiple myeloma. Br J Haematol. (2005) 131:71–3. doi: 10.1111/j.1365-2141.2005.05733.x. PMID: 16173965

[121] Mateos MV Richardson PG Schlag R Khuageva NK Dimopoulos M Shpilberg O . Bortezomib plus melphalan and prednisone compared with melphalan and prednisone in previously untreated multiple myeloma: updated follow-up and impact of subsequent therapy in the phase III VISTA trial. J Clin Oncol. (2010) 28:2259–66. doi: 10.1200/JCO.2009.26.0638. PMID: 20368561

[122] Delforge M Terpos E Richardson PG Shpilberg O Khuageva NK Schlag R . Fewer bone disease events, improvement in bone remodeling, and evidence of bone healing with bortezomib plus melphalan-prednisone vs. melphalan-prednisone in the phase III VISTA trial in multiple myeloma. Eur J Haematol. (2011) 86:372–84. doi: 10.1111/j.1600-0609.2011.01599.x. PMID: 21366694

[123] Qiang YW Barlogie B Rudikoff S Shaughnessy JD . Dkk1-induced inhibition of Wnt signaling in osteoblast differentiation is an underlying mechanism of bone loss in multiple myeloma. Bone. (2008) 42:669–80. doi: 10.1016/j.bone.2007.12.006. PMID: 18294945

[124] Terpos E Christoulas D Kastritis E Roussou M Migkou M Eleutherakis-Papaiakovou E . VTD consolidation, without bisphosphonates, reduces bone resorption and is associated with a very low incidence of skeletal-related events in myeloma patients post ASCT. Leukemia. (2014) 28:928–34. doi: 10.1038/leu.2013.267. PMID: 24045498

[125] Li Y Li J Zhuang W Wang Q Ge X Zhang X . Carfilzomib promotes the osteogenic differentiation potential of mesenchymal stem cells derived from myeloma patients by inhibiting notch1 activity *in vitro*. Leuk Res. (2014) 38:970–6. doi: 10.1016/j.leukres.2014.05.022. PMID: 24939218

[126] Terpos E Ntanasis-Stathopoulos I Katodritou E Kyrtsonis MC Douka V Spanoudakis E . Carfilzomib improves bone metabolism in patients with advanced relapsed/refractory multiple myeloma: results of the CarMMa study. Cancers (Basel). (2021) 13:1257. doi: 10.3390/cancers13061257. PMID: 33809268 PMC7998249

[127] Tibullo D Longo A Vicario N Romano A Barbato A Di Rosa M . Ixazomib improves bone remodeling and counteracts sonic Hedgehog signaling inhibition mediated by myeloma cells. Cancers (Basel). (2020) 12:323. doi: 10.3390/cancers12020323. PMID: 32019102 PMC7073172

[128] Diaz-delCastillo M Gundesen MT Andersen CW Nielsen AL Møller HEH Vinholt PJ . Increased bone volume by ixazomib in multiple myeloma: 3-month results from an open label phase 2 study. J Bone Miner Res. (2023) 38:639–49. doi: 10.1002/jbmr.4807. PMID: 36970780

[129] Bolzoni M Storti P Bonomini S Todoerti K Guasco D Toscani D . Immunomodulatory drugs lenalidomide and pomalidomide inhibit multiple myeloma-induced osteoclast formation and the RANKL/OPG ratio in the myeloma microenvironment targeting the expression of adhesion molecules. Exp Hematol. (2013) 41:387–397.e1. doi: 10.1016/j.exphem.2012.11.005. PMID: 23178378

[130] Guo J Fei C Zhao Y Zhao S Zheng Q Su J . Lenalidomide restores the osteogenic differentiation of bone marrow mesenchymal stem cells from multiple myeloma patients via deactivating Notch signaling pathway. Oncotarget. (2017) 8:55405–21. doi: 10.18632/oncotarget.19265. PMID: 28903429 PMC5589668

[131] Terpos E Christoulas D Kastritis E Katodritou E Papatheodorou A Pouli A . The combination of lenalidomide and dexamethasone reduces bone resorption in responding patients with relapsed/refractory multiple myeloma but has no effect on bone formation: final results on 205 patients of the Greek myeloma study group. Am J Hematol. (2014) 89:34–40. doi: 10.1002/ajh.23577. PMID: 23983166

[132] Attal M Lauwers-Cances V Hulin C Leleu X Caillot D Escoffre M . Lenalidomide, bortezomib, and dexamethasone with transplantation for myeloma. N Engl J Med. (2017) 376:1311–20. doi: 10.1056/NEJMoa1611750. PMID: 28379796 PMC6201242

[133] Terpos E Ntanasis-Stathopoulos I Kastritis E Hatjiharissi E Katodritou E Eleutherakis-Papaiakovou E . Daratumumab improves bone turnover in relapsed/refractory multiple myeloma; phase 2 study "REBUILD. Cancers (Basel). (2022) 14:2768. doi: 10.3390/cancers14112768. PMID: 35681747 PMC9179322

[134] Anderson KC Alsina M Atanackovic D Biermann JS Chandler JC Costello C . Multiple myeloma, version 2.2016: clinical practice guidelines in oncology. J Natl Compr Canc Netw. (2015) 13:1398–435. doi: 10.6004/jnccn.2015.0167. PMID: 26553768 PMC4891187

[135] Anderson K Ismaila N Flynn PJ Halabi S Jagannath S Ogaily MS . Role of bone-modifying agents in multiple myeloma: American Society of Clinical Oncology clinical practice guideline update. J Clin Oncol. (2018) 36:812–8. doi: 10.1200/JCO.2017.76.6402. PMID: 29341831

[136] Coleman R Body JJ Aapro M Hadji P Herrstedt J ESMO Guidelines Working Group . Bone health in cancer patients: ESMO Clinical Practice Guidelines. Ann Oncol. (2014) 25:iii124–37. doi: 10.1093/annonc/mdu103. PMID: 24782453

[137] Kumar SK Callander NS Adekola K Anderson LD Baljevic M Baz R . NCCN clinical practice guidelines in oncology (NCCN guidelines®) for multiple myeloma v1.2026. National Comprehensive Cancer Network (2025). Available online at: https://NCCN.org (Accessed October 1, 2025).

[138] Terpos E Sezer O Croucher PI García-Sanz R Boccadoro M San Miguel J . The use of bisphosphonates in multiple myeloma: recommendations of an expert panel on behalf of the European Myeloma Network. Ann Oncol. (2009) 20:1303–17. doi: 10.1093/annonc/mdn796. PMID: 19465418

[139] National Library of Medicine (U.S.) . Narlumosbart compared with denosumab in patients with multiple myeloma bone disease identifier NCT06314698. Available online at: https://clinicaltrials.gov/study/NCT06314698?cond=Multiple%20Myeloma&intr=Narlumosbart&rank=1 (Accessed October 1, 2025).

[140] Dhillon S . Narlumosbart: first approval. Drugs. (2024) 84:105–9. doi: 10.1007/s40265-023-01985-3. PMID: 38112898

[141] Tominaga A Wada K Kato Y Nishi H Terayama Y Okazaki K . Early clinical effects, safety, and appropriate selection of bone markers in romosozumab treatment for osteoporosis patients: a 6-month study. Osteoporos Int. (2021) 32:653–61. doi: 10.1007/s00198-020-05639-y. PMID: 32979066

[142] Liu L Wu S Wei L Xia Z Ji J Huang D . Romosozumab adverse event profile: a pharmacovigilance analysis based on the FDA Adverse Event Reporting System (FAERS) from 2019 to 2023. Aging Clin Exp Res. (2025) 37:23. doi: 10.1007/s40520-024-02921-5. PMID: 39808360 PMC11732777

[143] National Library of Medicine (U.S.) . A study of romosozumab in women with multiple myeloma and osteoporosis indetifier NCT05775094. Available online at: https://clinicaltrials.gov/study/NCT05775094?cond=Multiple%20Myeloma&intr=Romosozumab%20&rank=1A (Accessed October 1, 2025).

[144] Fulciniti M Tassone P Hideshima T Vallet S Nanjappa P Ettenberg SA . Anti-DKK1 mAb (BHQ880) as a potential therapeutic agent for multiple myeloma. Blood. (2009) 114:371–9. doi: 10.1182/blood-2008-11-191577. PMID: 19417213 PMC2714212

[145] Iyer SP Beck JT Stewart AK Shah J Kelly KR Isaacs R . A phase IB multicentre dose-determination study of BHQ880 in combination with anti-myeloma therapy and zoledronic acid in patients with relapsed or refractory multiple myeloma and prior skeletal-related events. Br J Haematol. (2014) 167:366–75. doi: 10.1111/bjh.13056. PMID: 25139740

[146] National Library of Medicine (U.S.) . A double-blind, placebo-controlled, randomized phase 2 study of BHQ880, an anti-Dickkopf1 (DKK1) monoclonal antibody (mAb), in patients with untreated multiple myeloma and renal insufficiency indentifier NCT01337752. Available online at: https://clinicaltrials.gov/study/NCT01337752 (Accessed October 1, 2025).

[147] National Library of Medicine (U.S.) . A single-arm, open-label, phase 2 clinical trial evaluating disease response following treatment with intravenous BHQ880, a fully human, anti-Dickkopf1 (DKK1) neutralizing antibody in previously untreated patients with high-risk, smoldering multiple myeloma indetifier NCT01337752. Available online at: https://clinicaltrials.gov/study/NCT01302886 (Accessed October 1, 2025).

[148] Abdulkadyrov KM Salogub GN Khuazheva N Shah J Kelly KR Isaacs R . Sotatercept in patients with osteolytic lesions of multiple myeloma. Br J Haematol. (2014) 165:814–23. doi: 10.1111/bjh.12835. PMID: 24650009 PMC4312883

[149] National Library of Medicine (U.S.) . Sotatercept (ACE-011) with lenalidomide or pomalidomide and dexamethasone in relapsed/refractory multiple myeloma identifier NCT01562405. Available online at: https://clinicaltrials.gov/study/NCT01562405 (Accessed October 1, 2025).

[150] Diamond TH Golombick T Manoharan A Ramakrishna R Bryant C . Teriparatide (recombinant human parathyroid hormone 1-34) therapy in myeloma patients with severe osteoporosis and fractures despite effective anti-myeloma therapy and bisphosphonates: a pilot study. Am J Hematol. (2020) 95:E272–6. doi: 10.1002/ajh.25919. PMID: 32602126

[151] Pirih FQ Michalski MN Cho SW Koh AJ Berry JE Ghaname E . Parathyroid hormone mediates hematopoietic cell expansion through interleukin-6. PloS One. (2010) 5:e13657. doi: 10.1371/journal.pone.0013657. PMID: 21048959 PMC2965090

[152] Bagni G Biancalana E Chiara E Costanzo I Malandrino D Lastraioli E . Epigenetics in autoimmune diseases: Unraveling the hidden regulators of immune dysregulation. Autoimmun Rev. (2025) 30:103784. doi: 10.1016/j.autrev.2025.103784. PMID: 40043893

[153] Hasani F Masrour M Khamaki S Hosseini S Heidarpour H Namazee M . Diagnostic and prognostic accuracy of MiRNAs in pancreatic cancer: a systematic review and meta-analysis. J Cell Mol Med. (2025) 29:e70337. doi: 10.1111/jcmm.70337. PMID: 39855897 PMC11761000

[154] Mihai AM Ianculescu LM Suciu N . MiRNAs as potential biomarkers in early breast cancer detection: a systematic review. J Med Life. (2024) 17:549–54. doi: 10.25122/jml-2024-0322. PMID: 39296436 PMC11407494

[155] Xu J Pan L Wu D Yao L Jiang W Min J . Comparison of the diagnostic value of various microRNAs in blood for colorectal cancer: a systematic review and network meta-analysis. BMC Cancer. (2024) 24:770. doi: 10.1186/s12885-024-12528-8. PMID: 38926893 PMC11209970

[156] Saliminejad K Khorram Khorshid HR Soleymani Fard S Ghaffari SH . An overview of microRNAs: Biology, functions, therapeutics, and analysis methods. J Cell Physiol. (2019) 234:5451–65. doi: 10.1002/jcp.27486. PMID: 30471116

[157] Hong DS Kang YK Borad M Sachdev J Ejadi S Lim HY . Phase 1 study of MRX34, a liposomal miR-34a mimic, in patients with advanced solid tumours. Br J Cancer. (2020) 122:1630–7. doi: 10.1038/s41416-020-0802-1. PMID: 32238921 PMC7251107

[158] Yehia AM Elsakka EGE Abulsoud AI Abdelmaksoud NM Elshafei A Elkhawaga SY . Decoding the role of miRNAs in multiple myeloma pathogenesis: a focus on signaling pathways. Pathol Res Pract. (2023) 248:154715. doi: 10.1016/j.prp.2023.154715. PMID: 37517169

[159] Katiyar A Kaur G Rani L Jena L Singh H Kumar L . Genome-wide identification of potential biomarkers in multiple myeloma using meta-analysis of mRNA and miRNA expression data. Sci Rep. (2021) 11:10957. doi: 10.1038/s41598-021-90424-y. PMID: 34040057 PMC8154993

[160] Rossi M Pitari MR Amodio N Di Martino MT Conforti F Leone E . miR-29b negatively regulates human osteoclastic cell differentiation and function: implications for the treatment of multiple myeloma-related bone disease. J Cell Physiol. (2013) 228:1506–15. doi: 10.1002/jcp.24306. PMID: 23254643

[161] Reagan MR Mishima Y Glavey SV Zhang Y Manier S Lu ZN . Investigating osteogenic differentiation in multiple myeloma using a novel 3D bone marrow niche model. Blood. (2014) 124:3250–9. doi: 10.1182/blood-2014-02-558007. PMID: 25205118 PMC4239334

[162] Wu S He X Li M Zhang Y Manier S Lu ZN . MiRNA-34a overexpression inhibits multiple myeloma cancer stem cell growth in mice by suppressing TGIF2. Am J Transl Res. (2016) 8(12):5433–5443. PMC5209494 28078014

[163] Moura SR Abreu H Cunha C Ribeiro-MaChado C Oliveira C Barbosa MA . Circulating microRNAs correlate with multiple myeloma and skeletal osteolytic lesions. Cancers (Basel). (2021) 13:5258. doi: 10.3390/cancers13215258. PMID: 34771422 PMC8582565

[164] Papanota AM Karousi P Kontos CK Artemaki PI Liacos CI Papadimitriou MA . A cancer-related microRNA signature shows biomarker utility in multiple myeloma. Int J Mol Sci. (2021) 22:13144. doi: 10.3390/ijms222313144. PMID: 34884950 PMC8658678

[165] Papanota AM Karousi P Kontos CK Ntanasis-Stathopoulos I Scorilas A Terpos E . Multiple myeloma bone disease: implication of microRNAs in its molecular background. Int J Mol Sci. (2021) 22:2375. doi: 10.3390/ijms22052375. PMID: 33673480 PMC7956742

[166] Raimondo S Urzì O Conigliaro A Bosco GL Parisi S Carlisi M . Extracellular vesicle microRNAs contribute to the osteogenic inhibition of mesenchymal stem cells in multiple myeloma. Cancers (Basel). (2020) 12:449. doi: 10.3390/cancers12020449. PMID: 32075123 PMC7072478

[167] McKeage K Plosker GL . Zoledronic acid: a pharmacoeconomic review of its use in the management of bone metastases. Pharmacoeconomics. (2008) 26:251–68. doi: 10.2165/00019053-200826030-00007. PMID: 18282018

[168] The Pharmaceutical Benefit Scheme . Pamidronate sodium. Available online at: https://www.pbs.gov.au/medicine/item/5669J-6288Y (Accessed October 1, 2025).

[169] Drugs.com . Xgeva prices, coupons, copay cards & patient assistance. Available online at: https://www.drugs.com/price-guide/xgeva#:~:text=The%20cost%20for%20Xgeva%20(120,not%20valid%20with%20insurance%20plans (Accessed October 1, 2025).

[170] Raje N Roodman GD Willenbacher W Shimizu K García-Sanz R Terpos E . A cost-effectiveness analysis of denosumab for the prevention of skeletal-related events in patients with multiple myeloma in the United States of America. J Med Econ. (2018) 21:525–36. doi: 10.1080/13696998.2018.1445634. PMID: 29480139

[171] Terpos E Jamotte A Christodoulopoulou A Campioni M Bhowmik D Kennedy L . A cost-effectiveness analysis of denosumab for the prevention of skeletal-related events in patients with multiple myeloma in four European countries: Austria, Belgium, Greece, and Italy. J Med Econ. (2019) 22:766–76. doi: 10.1080/13696998.2019.1606002. PMID: 30969797

[172] National Institute for Health Care and Excellence . Denosumab for preventing skeletal-related events in multiple myeloma (terminated appraisal). Available online at: https://www.nice.org.uk/guidance/ta549 (Accessed October 1, 2025). 41564218

[173] The Pharmaceutical Benefit Scheme . Denosumab. Available online at: https://www.pbs.gov.au/medicine/item/10061M-5110Y (Accessed October 1, 2025).

[174] Vogg B Poetzl J El Galta R Fuhr R Schwebig A Sekhar S . Pharmacokinetics and pharmacodynamics of the proposed biosimilar denosumab GP2411 and reference denosumab in healthy males. Expert Opin Biol Ther. (2024) 24:91–100. doi: 10.1080/14712598.2024.2308645. PMID: 38269652

[175] FDA . Denosumab. Wyost label. Available online at: https://www.accessdata.fda.gov/drugsatfda_docs/label/2024/761362s000lbl.pdf (Accessed October 1, 2025).

[176] Kim A Hong JH Shin W Yoo H Jung JG Reginster JY . A randomized, double-blind, single-dose, phase 1 study comparing the pharmacokinetics, pharmacodynamics, safety, and immunogenicity of denosumab biosimilar CT-P41 and reference denosumab in healthy males. Expert Opin Biol Ther. (2024) 24:655–63. doi: 10.1080/14712598.2024.2316846. PMID: 38349618

[177] Celltrion . Celltrion receives U.S. FDA approval for STOBOCLO® (denosumab-bmwo) and OSENVELT® (denosumab-bmwo) biosimilars referencing PROLIA® and XGEVA®. Available online at: https://www.celltrion.com/en-us/company/media-center/press-release/3768 (Accessed October 1, 2025).

[178] Lee HA Kim S Seo H Kim S . A phase I, randomized, double-blind, single-dose pharmacokinetic study to evaluate the biosimilarity of SB16 (proposed denosumab biosimilar) with reference denosumab in healthy male subjects. Expert Opin Investig Drugs. (2023) 32:959–66. doi: 10.1080/13543784.2023.2273510. PMID: 37870163

[179] AJMC . FDA, EMA approve second pair of denosumab biosimilars. Available online at: https://www.centerforbiosimilars.com/view/fda-ema-approve-second-pair-of-denosumab-biosimilars (Accessed October 1, 2025).

[180] Monga A Gagan Jamwal P Sharma S Kaur A . Biosimilars: a critical review of development, regulatory landscape, and clinical implications. AAPS PharmSciTech. (2025) 26:46. doi: 10.1208/s12249-025-03038-2. PMID: 39870890

[181] Jeka S Dokoupilová E Kivitz A Żuchowski P Vogg B Krivtsova N . Equivalence trial of proposed denosumab biosimilar GP2411 and reference denosumab in postmenopausal osteoporosis: the ROSALIA study. J Bone Miner Res. (2024) 39:202–10. doi: 10.1093/jbmr/zjae016. PMID: 38477751 PMC11338045

[182] Reginster JY Czerwinski E Wilk K Borowy P Strzelecka A Budlewski T . Efficacy and safety of candidate biosimilar CT-P41 versus reference denosumab: a double-blind, randomized, active-controlled, phase 3 trial in postmenopausal women with osteoporosis. Osteoporos Int. (2024) 35:1919–30. doi: 10.1007/s00198-024-07161-x. PMID: 39042292 PMC11499533

[183] Chung YS Langdahl B Plebanski R Czerwinski E Dokoupilova E Supronik J . SB16 versus reference denosumab in postmenopausal women with osteoporosis: 18-month outcomes of a phase III randomized clinical trial. Bone. (2025) 192:117371. doi: 10.1016/j.bone.2024.117371. PMID: 39674388

[184] Raghunathachar SK Krishnamurthy KP Gopalaiah LM Abhijith D Prashant A Parichay SR . Navigating the clinical landscape: update on the diagnostic and prognostic biomarkers in multiple myeloma. Mol Biol Rep. (2024) 51:972. doi: 10.1007/s11033-024-09892-w. PMID: 39249557

